# Single‐Cell Atlas of Human Ovaries Reveals The Role Of The Pyroptotic Macrophage in Ovarian Aging

**DOI:** 10.1002/advs.202305175

**Published:** 2023-11-30

**Authors:** Chuanchuan Zhou, Qi Guo, Jiayu Lin, Meng Wang, Zhi Zeng, Yujie Li, Xiaolan Li, Yuting Xiang, Qiqi Liang, Jiawen Liu, Taibao Wu, Yanyan Zeng, Shanyang He, Sanfeng Wang, Haitao Zeng, Xiaoyan Liang

**Affiliations:** ^1^ Center of Reproductive Medicine The Sixth Affiliated Hospital Sun Yat‐sen University Guangzhou Guangdong 510080 China; ^2^ GuangDong Engineering Technology Research Center of Fertility Preservation Guangzhou Guangdong 510080 China; ^3^ Biomedical Innovation Center The Sixth Affiliated Hospital Sun Yat‐sen University Guangzhou Guangdong 510080 China; ^4^ Department of Obstetrics and Gynaecology Li Ka Shing Faculty of Medicine The University of Hong Kong Hong Kong S.A.R. 999077 China; ^5^ Reproductive Medicine Center The First People's Hospital of Foshan Foshan 528000 China; ^6^ Department of Obstetrics and Gynecology Affiliated Dongguan Hospital Southern Medical University Dongguan 523795 China; ^7^ Department of Gynecology Guangdong Provincial People's Hospital (Guangdong Academy of Medical Sciences) Southern Medical University Guangzhou 519041 China; ^8^ Department of Gynecology Guangdong Women and Children Hospital 521 Xing Nan Road Guangzhou Guangdong 511400 China

**Keywords:** aging, cell death, developmental trajectory, immune cells, intercellular communication, macrophage, ovary, pyroptosis, senescence, single‐cell RNA sequencing

## Abstract

Female fecundity declines in a nonlinear manner with age during the reproductive years, even as ovulatory cycles continue, which reduces female fertility, disrupts metabolic homeostasis, and eventually induces various chronic diseases. Despite this, the aging‐related cellular and molecular changes in human ovaries that occur during these reproductive years have not been elucidated. Here, single‐cell RNA sequencing (scRNA‐seq) of human ovaries is performed from different childbearing ages and reveals that the activation of the pyroptosis pathway increased with age, mainly in macrophages. The enrichment of pyroptotic macrophages leads to a switch from a tissue‐resident macrophage (TRM)‐involve immunoregulatory microenvironment in young ovaries to a pyroptotic monocyte‐derived macrophage (MDM)‐involved proinflammatory microenvironment in middle‐aged ovaries. This remolded ovarian immuno‐microenvironment further promotes stromal cell senescence and accelerated reproductive decline. This hypothesis is validated in a series of cell and animal experiments using GSDMD‐KO mice. In conclusion, the work expands the current understanding of the ovarian aging process by illustrating a pyroptotic macrophage‐involved immune mechanism, which has important implications for the development of novel strategies to delay senescence and promote reproductive health.

## Graphical abstract

ScRNA‐seq analysis of human ovaries identified age‐related changes in cellular interactomes during reproductive years.The immune regulatory mechanisms of ovarian aging are underscored by emphasizing the central role of pyroptotic macrophages in accelerating collagen deposition and stromal cell senescence in elder ovaries, which eventually lead to ovarian function decline.

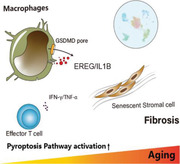

## 1 Introduction

Aging is a natural process accompanied by disease and imbalance of metabolic homeostasis. However, the process of aging is not synchronized across different organs, and each organ exhibits distinct patterns of aging.^[^ ^1^ ^]^ As a female endocrine organ, the ovaries appear to exhibit greater susceptibility to aging than other organs, culminating in menopause around the age of 50 as the reproductive lifespan ends.^[^ ^2^, ^3^ ^]^ The onset of menopause results in the loss of fertility and significantly impacts endocrine homeostasis, subsequently leading to a higher risk of cardiovascular complications and osteoporosis. Despite numerous reports based on animal experiments, the process of ovarian aging remains poorly understood. This is partly due to significant variations in aging rates among different species, and the aging rate may vary significantly between human and rodent species, and even nonhuman primates (NHPs).^[^ ^4^ ^]^ More importantly, recent studies have primarily focused on the disparities between young and fully aged‐ovaries. Yet, it is worth noting that women typically experience a non‐linear decline in fertility related to aging just prior to menopause.^[^ ^5^, ^6^ ^]^ Human ovarian aging is not limited to menopause, as ovarian function and fertility decline significantly after the age of 35,^[^ ^7^ ^]^ with menopause often being a consequence of this process. However, the crucial underlying changes that occur prior to menopause have not been adequately defined. Consequently, these issues remain of great interest in the field of ovarian aging biology.

Ovarian aging is a complicated process.^[^ ^8^ ^]^ Inflammatory aging is a crucial characteristic of organ senescence. Similarly, within the ovary, an established hallmark of ovarian aging is the emergence of an inflammatory phenotype, which involves elevated expression levels of pro‐inflammatory cytokines, collagen deposition and a dysfunctional immune system.^[^ ^9^, ^10^ ^]^ Recent studies in mice have provided evidence of how the NLRP3 (NOD‐like receptor family pyrin domain‐containing 3) inflammasome modulates ovarian aging^[^ ^11^, ^12^ ^]^ and how anti‐inflammatory agents, such as melatonin,^[^ ^13^ ^]^ metformin and resveratrol,^[^ ^13^, ^14^ ^]^ delay senescence. Thus, inflammation has been hypothesized to be a major cause of ovarian aging. Macrophages, the central components of the inflammatory immune response, are the most abundant immune cells in the ovary, and perform multiple functions to maintain tissue homeostasis.^[^ ^8^, ^15^ ^]^ A strong connection between aberrant immunity and ovarian aging has been referenced in a recently published transcriptomic profile of young and aged murine ovaries.^[^ ^16^ ^]^ The balance between monocyte‐derived and tissue‐resident macrophages is perturbed in aged ovaries, and this shift in macrophage polarization contributes to the aging process. These results emphasize the critical role of inflammation regulation by immune cells in the aging process. However, considering the extensive heterogeneity in the ontogeny, tissue‐specific phenotypes and functions of macrophage populations, the age‐related changes in the ovarian immune microenvironment was not accurately and meaningfully delineated in this study, due to the limitation of conventional bulk RNA‐seq and the inapplicability of the classic M1/M2 model.

Recently, advances such as single‐cell RNA sequencing (scRNA‐seq) technologies have provided a novel cell atlas of normal adult human ovaries and deepened our understanding of the molecular basis of follicle remodeling. The single‐cell atlas of human ovarian tissue composed of follicles and surrounding tissues demonstrates the fundamental cellular composition and provides insights into the relationship between follicular development and ovarian microenvironment.^[^ ^17^ ^]^ Magdalena Wagner has highlighted that the existence of functional germline stem cells in postnatal human ovaries, which has long remained controversial,^[^ ^18^, ^19^ ^]^ is more like a fantasy. ScRNA‐seq of human ovaries provides compelling evidence for the investigation of ovarian germ stem cells. While their existence is indisputable, this also lends support to the issue of DDX4 antibody selection in germ stem cell research.^[^ ^20^ ^]^ In (STRT‐seq) analysis of young and aged NHPs, the increased levels of reactive oxygen species (ROS) and disturbed antioxidant pathways in oocytes and granulosa cells have been confirmed to account for ovarian function decline with age.^[^ ^21^ ^]^ However, precise scRNA‐seq is lacking to explain the changes in cell subpopulations and the inflammatory aging characteristics of the immune microenvironment during human ovarian aging, especially during the reproductive lifespan. We strongly believe that uncovering the underlying alternations in human ovaries throughout the finite reproductive span at a single‐cell resolution could yield further valuable insights into the ovarian aging process and clinical references.

Therefore, in our work, we used single‐cell transcriptomics to comprehensively identify the aging‐related changes in cellular composition and the distinct genetic signatures in human adult ovaries and unveiled the immune‐modulation mechanisms that participated in the shift of cellular state and developmental trajectory of ovarian cells. We further carried out a ligand‐receptor pair analysis to delineate the complex macrophage‐centered intercellular networks. Finally, our breakthrough in scRNA‐seq findings was verified by ex vivo cell experiments and studies in GSDMD‐KO mice. Overall, our results provide insights into the pivotal roles of immune cells, especially macrophages, in ovarian aging and will help to develop potential strategies to slow the aging process and prolong female reproductive health.

## 2 Results

### 2.1 Ovarian Function is Declined in Middle‐Aged Women

**Figure 1:**
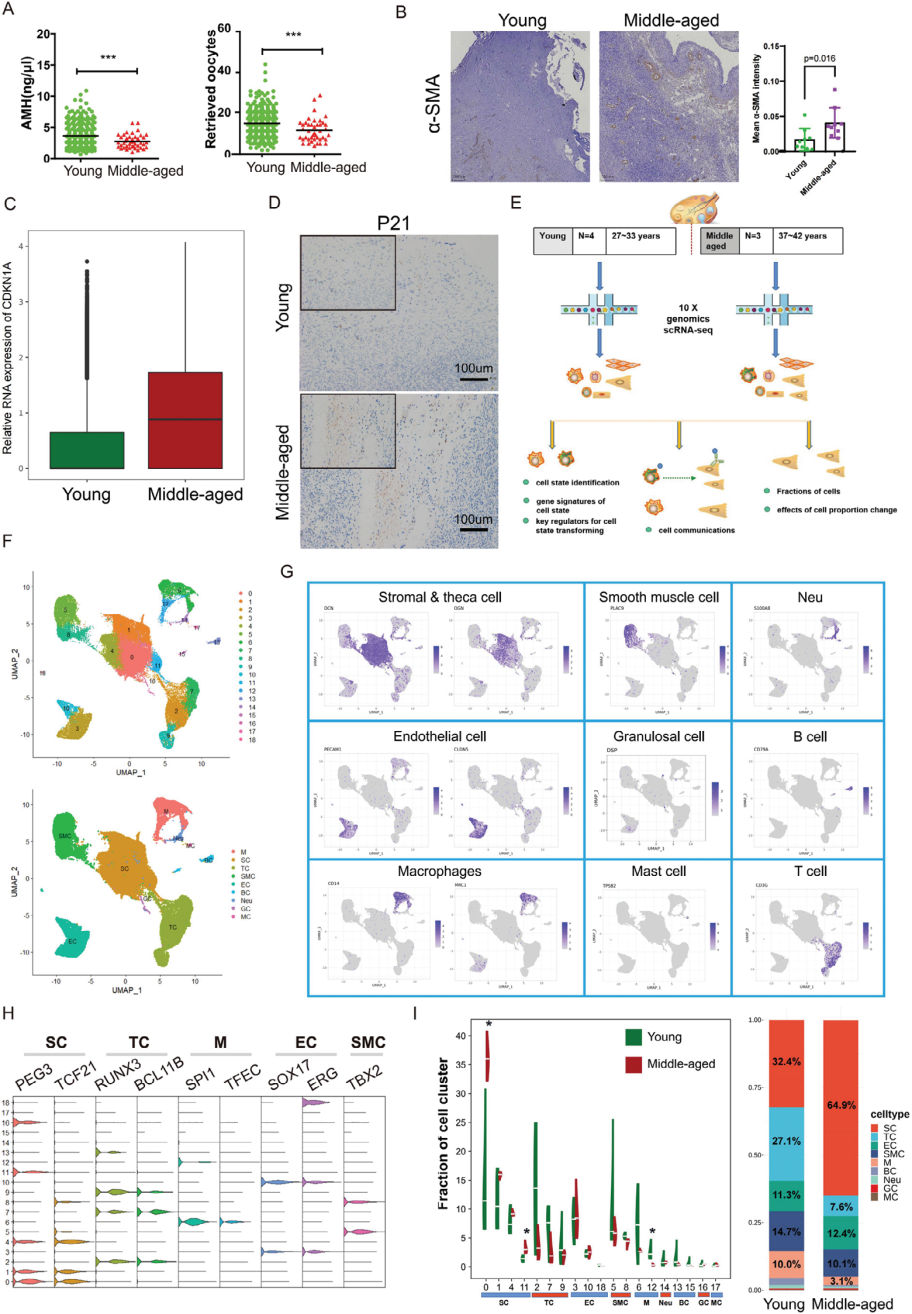
Single‐cell transcriptome map of reproductive‐age human adult ovaries. A) Serum AMH levels and number of retrieved oocytes for young and middle‐aged women. B) Representative α‐SMA IHC staining of ovarian sections from young and middle‐aged women, and the collagen fibers were dyed blue. The fibrosis area was calculated as the area of fibrosis to the total area. A total of 30 sections from 10 individuals were analyzed and the corresponding statistical results were showed in the right. C) Histogram presenting the relative RNA expression of CDKN1A in young and middle‐aged women. D) Representative IHC staining for P21 showing the elevated expression of P21 in middle‐aged group. Bar: 100um. The enlarged images were showed on the opt left corner of the graph. E) Schematic illustration of the experimental workflow for single‐cell analysis of human ovaries. F) UMAP plots showing the distribution of the 9 major specific ovarian somatic cell populations and 19 clusters; each dot represents a single cell. G) UMAP plots showing the expression levels of selected canonical marker genes in each cell type. The color of the dot indicates the gene expression intensity. H) Violin plots indicating the expression levels of representative transcriptional regulators in different cell types. I) Bar graphs represent the composition ratio of each cell type from young (n = 4) and middle‐aged (n = 3) women. Boxplots showing the proportion of each cluster from young (n = 4) and middle‐aged (n = 3) women.

It is well known that fertility diminishes in women over the age of 35 years, as reflected by the decreased quality and quantity of oocytes.^[^ ^5^, ^22^ ^]^ We retrospectively analyzed 347 women admitted to our reproductive center due to male factor infertility and divided them into two groups based on age: < 33 years (young group) and > 37 years (middle‐aged group). The declined ovarian function in the middle‐aged group was confirmed by decreased anti‐Mullerian hormone (AMH) and lower number of retrieved oocytes (Figure 1A ). Moreover, we found significantly increased ovarian fibrosis in the middle‐aged group (**Figure** **1**B; Figure S1A, Supporting Information), which suggested that age‐associated differences of ovarian microenvironment and function were already present in reproductive age.^[^ ^22^, ^23^ ^]^ Ovarian aging precedes somatic aging, however, the underlying mechanisms of ovarian function loss in reproductive age are largely unknown.

Since the age gap between the two groups in our study was not as significant as that in traditional aging research, the expression of aging‐related markers in the CellAge datebase (https://genomics.senescence.info/cells/) was evaluated among all the sequencing individuals^[^ ^24^ ^]^ (Figure S1B, Supporting Information). The canonical marker for organismal aging, *CDKN1A*, was upregulted at RNA level in middle‐aged group (Figure 1C). Consistently, the expression level of corresponding protein (P21) was also increased in middle‐aged ovaries (Figure 1D; Figure S1C, Supporting Information). This indicated that prior to menopause, when the chronological age has not yet fully declined, the ovaries exhibit aging signs. Thus, we aim to investigate the mechanisms underlying ovarian aging during this premenopausal stage.

### 2.2 ScRNA‐seq Reveals A Diverse Ovarian Microenvironment

To explore the changes in ovarian microenvironment at single‐cell resolution and comprehensively unravel the mechanisms of age‐related ovarian function decline during the reproductive period, we collected ovarian tissues from 4 young patients (aged 27, 27, 29, and 33 years) and 3 middle‐aged patients (aged 37, 42, and 42 years). The representative histological figures from the two groups, stained by H&E, are presented in Figure S1D (Supporting Information). A single cell suspension was prepared by enzymatic dissociation (visible follicles were removed) and profiled by scRNA‐seq (Figure 1E). Similar cellular heterogeneity was observed between each sample (Figure S1E, Supporting Information). Thus, all 7 samples were combined for downstream analysis.

Unbiased clustering analysis revealed 19 distinct cell types in the ovaries. For cell type annotation, we compared the differentially expressed genes (DEGs) in each cluster with the marker genes from the previously published ovarian scRNA‐seq datasets and Human Cell Atlas datasets. Nine major clusters were corroborated: stromal cells (SCs, cluster [CL] 0, 1, 4, 11), T cells (TCs, CL2, 7, 9), endothelial cells (ECs, CL3, 10, 18), macrophages (Ms, CL6, 12), neutrophil (Neu, CL14), smooth muscle cells (SMCs, CL5, 8), B cells (BCs, CL13,15), granulosa cells (GCs, CL16), and mast cells (MCs, CL17) (Figure 1F). Each cell type had a distinct gene expression profile. The representative marker genes for each cluster were shown in the uniform manifold approximation and projection (UMAP) cluster map (Figure 1G) and the specific transcription factors in five major clusters were identified and presented as violin plots (Figure 1H).

While cells from all the identified clusters were presented in both age groups, the relative frequency of each cell type differed significantly. The proportion of SCs showed a general increase in the middle‐aged group, especially CL0 and CL11. In contrast, Ms and TCs showed a significant decline with age, especially CL12 in Ms. The proportions of other cell types such as ECs (CL3, 10, 18), SMCs (CL5, 8), BC (CL13, 15) and GCs (CL16) were approximately similar between the two groups (Figure 1I).

### 2.3 Senescent SCs Accumulate in Middle‐Aged Ovaries

**Figure 2:**
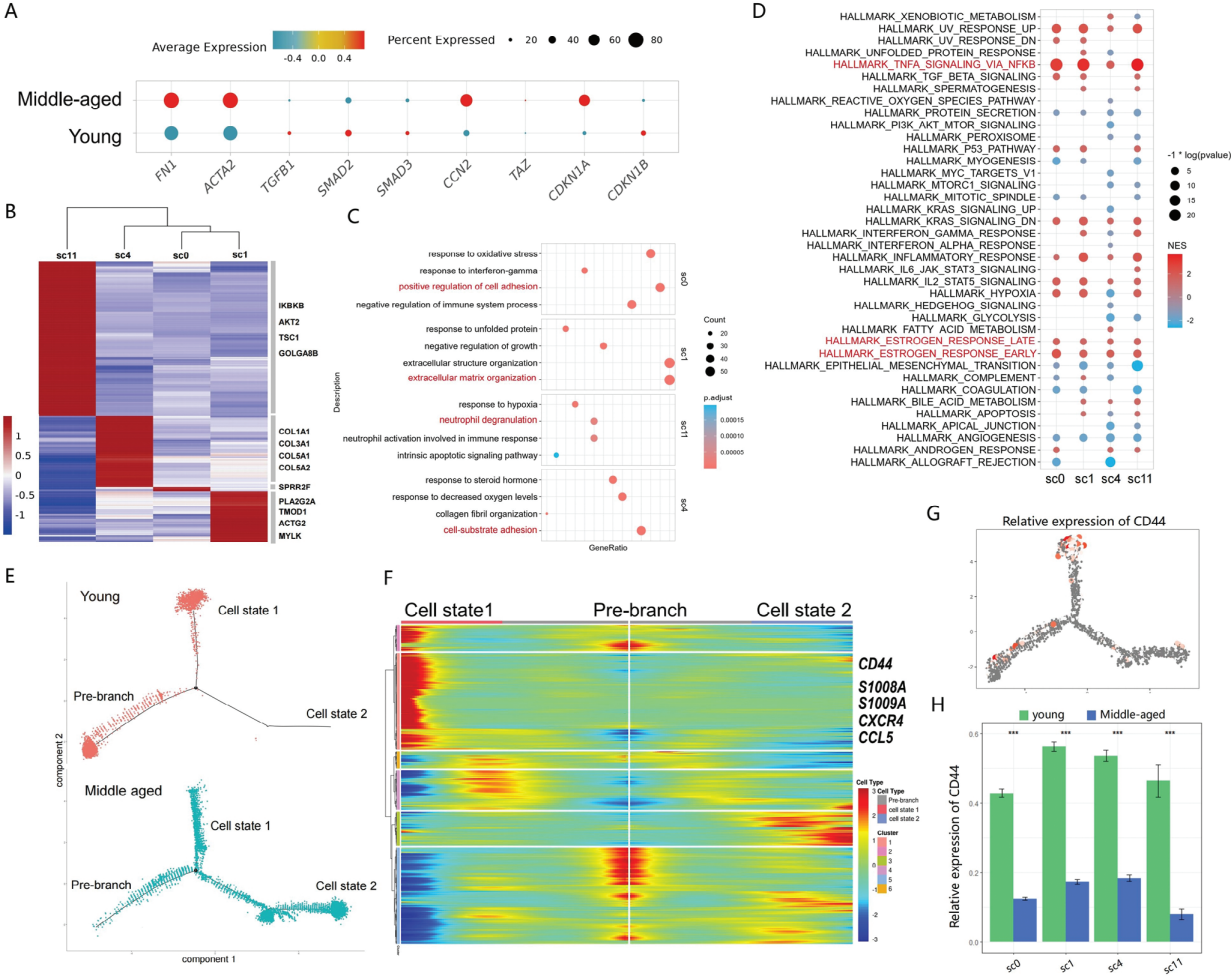
Senescence of stromal cells increased with age. A) Bubble plots of the expression of fibrosis‐related and senescence‐related genes. Bubble sizes are proportional to the number of positive cells and color represents relative expression levels. B) Heatmap showing differential gene expression in four stromal cell subtypes. The canonical markers used to identify each subtype are plotted. C) Bubble plots of GO enrichment results for age‐related DEGs in sc0, sc1, sc4, and sc11. D) Bubble plots of GSEA results. The color of the bubble reflects the normalized enrichment score (NES), and bubble size indicates the significance of the enrichment. E) Comparison of the distribution of stromal cells (sc0) from the young and middle‐aged groups along the pseudotime trajectories. F) Heatmap illustrating the differentially expressed genes toward cell state 1 and cell state 2 along the pseudotime trajectories. G) Gene expression pattern of CD44 along the pseudotime trajectories. Both dot size and dot color represent the expression level of CD44. H) Histogram representing the relative expression of CD44 in each stromal cell cluster in young and middle‐aged women.

SCs, the principal component of the ovarian stroma, can differentiate into inner TCs and outer myofibroblasts, the major extracellular matrix (ECM)‐producing cells in the ovarian stroma.^[^ ^23^, ^25^ ^]^ Given their pivotal roles in collagen deposition, the expression of fibrosis‐related genes in SCs, such as *FN1*, *ACTA2*, and *CCN2* was first compared between two groups (**Figure** **2**A). Consistent with the increased ovarian fibrosis, the proportion of SCs and the expression of fibrosis‐related genes were elevated in middle‐aged group. We subsequently sought to delineate the characteristics of different SC subtypes and their relationships with age. The marker genes of 4 separate SC clusters (sc0, 1, 4, 11) were identified. Sc1, with high *PLA2G2A* expression, was related to adhesion and structure. Sc4, characterized by the collagen‐related genes *COL1A1*, *COL3A1*, and *COL5A1*, was involved in ECM formation (Figure 2B). The marker genes, *SPRR2F*, *PLA2G2A*, *COL5A1*, and *GOLGA8B* was highly expressed in sc0, sc1, sc4, and sc11, respectively (Figure S2A, Supporting Information).

To further explore the specific functional pattern of the age‐associated differentially expressed genes (DEGs), we filtered out and clustered DEGs in each subpopulation and then performed Gene Ontology (GO) analysis and gene set enrichment analysis (GSEA). The GO results of the aged‐related DEGs in each subcluster showed that “positive regulation of cell adhesion”, “extracellular matrix organization”, “neutrophil degranulation” and “cell‐substrate adhesion” were highly enriched in the middle‐aged group, suggesting the involvement of SCs, especially sc1, in the development of fibrosis in middle‐aged ovaries (Figure 2C). GSEA strongly suggested similar age‐related DEG patterns among 4 SC cell subclusters, with a significant enrichment in “TNFA_SIGNALING” and “ESTROGEN_RESPONSE” (Figure 2D).

Next, we applied pseudotime analysis within each SC subset to explore the age‐associated differences in cellular states. For example, sc0 could be classified into 3 states: pre‐branch, cell state 1, and cell state 2 (Figure 2E). The sc0 in the young group was predominantly in pre‐branch and cell state 1, while the proportion of cell state 2 was significantly increased in the middle‐aged sc0. BEAM analysis revealed that an expression pattern occupied by a series of immune regulatory genes such as *CD44*, *S100A8*, *S100A9*, and *CCL5*, which is highly and poorly expressed in cell state 1 and cell state 2, respectively (Figure 2F,G). *CD44* has been reported to be implicated in microenvironmental signal transduction. The transcription level of *CD44* exhibited age‐related decrease in both sc1 and other SC subclusters (Figure 2H). Actually, sc1 and sc4 shared similar age‐related cellular state distribution features as sc0 (Figure S2B, Supporting Information).

### 2.4 Ovarian Resident Macrophages Exhibit Extensive Heterogeneity and Middle‐Aged Ovaries are Dominated by Pyroptotic Macrophages

Macrophages, characterized by their substantial heterogeneity, plasticity and multifunctionality, have been reported to be the most abundant immune cells in ovaries, contributing to the normal physiological and pathological changes in the ovary.^[^ ^26^, ^27^ ^]^ Previous studies have indicated that ovarian macrophages are composed of a mixed population of tissue‐resident macrophages (TRMs), which derived from the extra‐embryonic yolk sac and fetal liver before birth, and monocyte‐derived macrophages (MDM), which derived from the bone marrow after birth. These two types of macrophages play distinct roles in maintaining tissue homeostasis. While TRMs clear senescent cells and extracellular products, MDMs are differentiated from recruited monocytes when inflammation occurs. However, in‐depth research on the phenotypes of ovarian macrophages is scarce.

**Figure 3:**
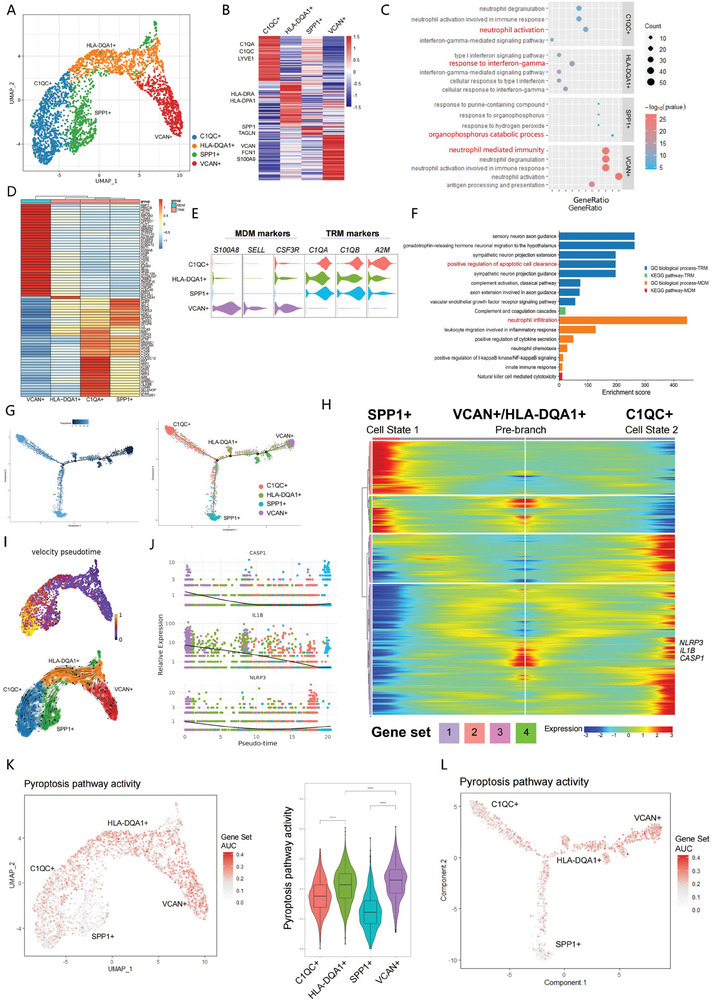
Characterization and cell trajectory analysis of macrophages in the ovaries. A) UMAP plots showing 4 monocyte/macrophage subclusters. B) Heatmap of marker genes of macrophage subclusters. C) GO terms enriched among the marker genes in each subcluster are shown in the bubble plot. D) Heatmap showing the expression of canonical marker genes of TRMs and MDMs in four monocyte/macrophage subtypes. MDMs, monocyte‐derived macrophages, TRMs, tissue‐resident macrophages. E) The canonical MDM and TRM markers are differentially expressed in each subcluster. F) GO and KEGG analyses of differentially expressed genes in TRMs and MDMs. G) Pseudotime analysis of monocyte/macrophage clusters. H) Heatmap illustrating branch‐specific DEGs dynamics along the pseudotime trajectory. Cell state 1 indicates SPP1+ TRM cells, and cell state 2 indicates C1QA+ TRM cells. I) RNA velocity analysis of macrophage clusters overlaid on the UMAP plot. Arrows represent the direction of pseudotime. J) The dynamic expression of *CASP1*, *IL1B*, and *NLRP3* along the pseudotime trajectory. K) AUC scores of pyroptosis pathway activity for individual cells. Violin plots show the pyroptosis pathway activity scores for each subcluster. L) The pyroptosis pathway activity scores projected onto the pseudotime trajectory to illustrate the relationship between pyroptosis pathway activity and macrophage differentiation.

Two major macrophage clusters were identified in our unsupervised cluster analysis, including CL6 (*LYZ* ^high^, *CTSS* ^high^, *S100A4* ^high^, *CD14* ^high^, *MS4A6A* ^high^) and CL12 (*SELENOP* ^high^, *APOE* ^high^, *GYPC* ^high^). This observation challenges the classic classification of macrophage, when we aligned our data with canonical markers in public databases and published literature, we found a mixed expression of reported markers of several distinct populations in a single cluster. For instance, CL6 was enriched in both M1 and M2 associated genes, indicating that the global clustering analysis is inadequate to resolve the heterogeneity of this cluster. Thus, we further grouped ovarian macrophages into 4 subclusters (subset 0–3), designated as subset 0 (C1QC+ macrophages), subset 1 (HLA‐DQA1+ macrophages), subset 2 (SPP1+ macrophages), subset 3 (VCAN+ macrophages) (**Figure** **3**A 1‐4). The expression of marker genes in each subset was compared and presented in a heatmap and violin plot (Figure 3B; Figure S3A, Supporting Information). GO analysis showed that the marker genes in each subcluster were mainly enriched in “neutrophil activation”, “response to interferon‐gamma”, “organophosphorus catabolic process” and “neutrophil mediated immunity”, respectively (Figure 3C). A canonical list of genes distinguishing TRMs and MDMs was applied to compare the gene expression profiles of each subclusters.^[^ ^28^ ^]^ This analysis revealed that C1QC+, HLA‐DQA1+, SPP1+ macrophages were TRMs, whereas VCAN+ macrophages were MDMs (Figure 3D). On this basis, we delineated the phenotypes of ovarian resident macrophages and described their specific markers in detail, since the characteristics of macrophages vary among tissues, largely depending on their location. In addition to the general high expression of *C1QA*, *C1QB*, *C1QC*, and *CD81* and low expression of *SELL*, *S100A8*, and *S100A9* in other TRM populations, *A2M* was specifically highly expressed and *CSF3R* was poorly expressed in ovarian TRM (Figure 3E). To further elucidate the functional heterogeneity of macrophage populations, we performed GO analysis, which revealed that MDMs were involved in biological processes related to neutrophil infiltration and inflammatory responses, while TRMs were enriched in apoptotic cell elimination (Figure 3F).

A similar macrophage classification approach was first proposed by Zhang.^[^ ^29^ ^]^ He suggested that SPP1+ and C1QC+ were highly expressed in two functionally and developmentally distinct tissue‐resident macrophages, respectively, and that these two cell types arose from the VCAN+ MDM, according to developmental trajectory analysis. Therefore, we subsequently performed pseudotime analysis (Figure 3G) and RNA velocity analysis (Figure 3I) to explore whether ovarian macrophages shared similar developmental trajectories with tumor‐associated macrophages. Subset 3 (VCAN+) lies at the root of the trajectory, and can differentiate into subset 1 (HLA‐DQA1+). Two distinct directions beyond subset 1 were populated by subset 0 (C1QC+) and subset 2 (SPP1+). We clustered the genes regulated at the bifurcation point into four distinct gene sets according to their expression patterns (Figure 3H). *IL1B*, *CASP1*, and *NLRP3* were markedly downregulated in the transition to cell state 1, but notably upregulated in the transition to cell state 2. Their dynamic expression is plotted along the pseudotime trajectory to show that the expression decreased gradually with pseudo‐time (Figure 3J). Meanwhile, the results of BEAM analysis showed that genes sharing this pattern (gene set 1), such as *IL1B*, *CASP1*, and *NLRP3*, were enriched in the process of pyroptosis, a newly discovered form of cell death that was first detected in macrophages. Thus, we scored the activity of pyroptosis pathways in individual cells by AuCell algorithm^[^ ^30^ ^]^ to explore the relationship between pyroptosis activation and cell state determination. Notably, the VCAN+ macrophage clusters were characterized by higher pyroptosis pathway activity (Figure 3K). Pyroptosis pathway activity declined with further macrophage subtype transition, as illustrated in Figure 3L. Thus, we propose that macrophages with high expression of pyroptosis‐related genes, mostly VCAN+ macrophages, have a propensity to undergo pyroptosis. However, whether pyroptosis activation is a causal factor or an accompanying consequence during macrophage subtype transition is poorly known.

**Figure 4:**
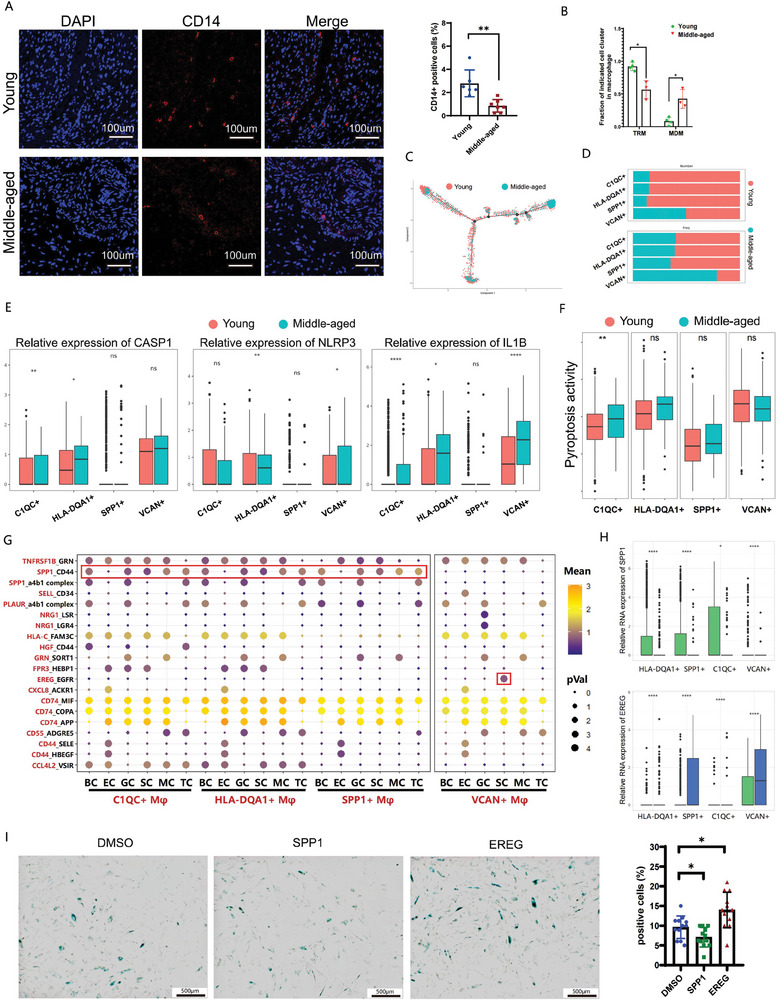
Age‐related changes in macrophages and subsequent impacts on ovarian microenvironment A) Immunostaining of CD14 in young (upper panel) and middle‐aged (lower panel) ovarian sections. Bar:100um. The number of CD14 positive cells was counted and displayed in the right statistical graph. B) Proportions of TRM and MDM subsets in the total macrophages in young and middle‐aged women. C) Pseudotime visualization of the distributions of macrophage populations in young and middle‐aged women. Each dot represents one cell. D) Bar plot illustrating the numbers and proportions of each macrophage subset in the young and middle‐aged groups. E) Boxplot diagram showing the relative expression of *CASP1*, *NLRP3* and *IL1B* in each macrophage subcluster from young and middle‐aged women. F) Boxplot diagram showing the scores of pyroptosis pathway activity in each macrophage cluster from the two groups. G) Bubble plots showing the mean strength of selected ligand‐receptor pairs in macrophage subclusters with other cell types. The dot size represents the p value. H) Boxplots plots showing the different expression of selected ligands in macrophage subclusters between the young and middle‐aged groups. I) Representative images of senescence‐associated β‐galactosidase (SA‐beta‐gal) staining of stromal cells treated with SPP1 and EGF. Statistical analyses of SA‐beta‐gal‐positive cell rates were shown in the right image.

To address this issue, the macrophage phenotypes of the two groups were compared. The total number of macrophages decreased in middle‐aged group, as indicated by CD14‐staining (**Figure** **4**A). More specifically, we found a markedly reduced proportion of TRMs in middle‐aged group, while that of MDMs remained unchanged, resulting in a decreased ratio of TRMs to MDMs in the middle‐aged group (Figure 4B). Thus, ovarian aging was associated with high proportions of MDMs and low proportions of TRMs, also confirmed by the differential expression of TRM and MDM marker genes between the two groups (Figure S3B, Supporting Information). The effects of aging on the macrophage subtype transition were also evaluated by mapping these cells along the pseudotemporal trajectory (Figure 4C). The proportions of C1QC+ and SPP1+ TRMs decreased significantly, while the VCAN+ MDMs increased (Figure 4D), indicating that the transition from VCAN+ to SPP1+/C1QC+ macrophages was inhibited in middle‐aged group.

Next, we clustered DEGs of each subcluster in the two groups and performed GSEA and GO analysis to elucidate age‐related changes. The GSEA and GO analyses revealed that immune modulation and inflammatory response pathways such as neutrophil activation in C1QC+ macrophages, type I interferon signaling pathway in HLA‐DQA1+ macrophages and neutrophil degranulation in VCAN+ macrophages, were significantly enriched in middle‐aged group (Figure S3C, Supporting Information). Meanwhile, the expression of pyroptosis‐related genes, such as *IL1B*, *CASP1*, and *NLRP3*, was significantly increased in VCAN+ MDMs from middle‐aged group (Figure 4E). Despite that, pyroptotic activity appeared to be functional characteristic of macrophage subsets and was exclusively elevated in VCAN+ macrophages. The pyroptosis activity of each subcluster was comparable between two groups (Figures 3K and 4F). In summary, macrophages characterized by high pyroptosis activity were accumulated in middle‐aged ovaries. Thus, we referred to it as pyroptotic macrophages to highlight this functional characteristic in our subsequent research.

### 2.5 The Number of Effector T Cells Increases With Advancing Age

T cells comprised subpopulations including *CCL* ^+^ *GZMA* ^+^ *AREG* ^+^ T cells (AREG T cells, cluster 0), *CD8* ^+^ *GZMK* ^+^ T cells (GZMK T cells, cluster 1), *FCGR3A* ^+^ *FGFBP2* ^+^ *GZMB* ^+^ *GNLY* ^+^ (effector T cells, cluster 2), *TCF7* ^+^ *CCR7* ^+^ T cells (naïve CD4 T cells, cluster 3), and *CD8* ^+^ *ZBTB16* ^+^ T cells (MAIT T cells, cluster 4), according to the second clustering results. The functional analysis of marker genes of each cluster suggested that T‐cell clusters in ovaries shared a high degree of taxonomic similarity to those in other tissues,^[^ ^31^, ^32^, ^33^ ^]^ with the same markers and similar functional gene sets (Figure S4A—C, Supporting Information). Furthermore, A differentiation trajectory from AREG T cells to GZMK T cells and effector T cells was generated by RNA velocity analysis (Figure S4D, Supporting Information). We then investigated the effects of aging on T cell subpopulations. The total number of T cells was decreased in middle‐aged ovaries, confirmed by CD3 immunofluorescence staining (Figure S4E, Supporting Information). In which, AREG T cell decreased, while effector T cell increased significantly (Figure S4F, Supporting Information). We performed GSEA analysis in each cluster to reveal the age effects on T‐cell functions and showed TNF‐α and IL6‐STAT3 pathways were activated in the middle‐aged group (Figure S4G, Supporting Information).

### 2.6 The Ovarian Interactomes Change Dramatically With Increasing Age

Having successfully deciphered the cellular composition and age‐related changes in cell subtypes and functions in the human ovary, we next aimed to explore the potential initiators or drivers of the aging process by constructing a comprehensive intercellular network. A heatmap and the network diagram were presented to illustrated the number of significant interactions among all the cell types, suggesting the intensity of intercellular communication. Macrophages interacted closely with other cells (Figure S5A,B, Supporting Information). Considering that the dominant macrophage subset changed with age and macrophage subtype with special functional phenotype accumulated in middle‐aged ovaries, we identified ligand‐receptor pairs in different macrophage subsets to explore their implications. EREG‐EGFR pairs, NRG1‐LSR pairs and SELL‐CD34 pairs were enriched between VCAN+ macrophages (Mφ) and other somatic cells, while C1QC+, HLA‐DQA1+ and SPP1+ macrophage (Mφ) communicated with other somatic cells mainly through SPP1 ligand (Figure 4G). We subsequently compared the expression profiles of ligands in macrophage subclusters from young and middle‐aged groups. Among them, *EREG*, *SELL*, and *PLAUR* were mainly expressed in VCAN+ macrophages, with a relatively higher expression in middle‐aged group. The expression of *SPP1*, *NRG1*, and *CD74* was higher in young group (Figure 4H; Figure S5C, Supporting Information). Their corresponding receptors, CD44 and EGFR, were ubiquitously expressed on TC and SCs. SPP1 has been reported to regulate T cell development, and T cells with SPP1 deficiency preferentially differentiate into cytotoxic CD8+ T cells,^[^ ^34^ ^]^ while its effects on SCs remained unknown. Therefore, we assessed the biological functions of SPP1 and EREG on SCs by senescence‐associated β‐galactosidase (SA‐beta‐gal) staining. Consistent with our expectations, SPP1 significantly countered SC senescence, and the opposite effects were observed following EREG treatment (Figure 4I).

These results suggested non‐pyroptotic macrophages served as an immune‐regulatory role in young ovaries via the secretion of SPP1, interacting with stromal cells to keep them in a younger state (cell state 1). While in the middle‐aged ovaries, the proportions of macrophage subclusters changed. Downregulated expression of SPP1 weakened the communication with SCs and T cells, promoting Effector‐T‐cell transition. The VCAN+ macrophages, with high pyroptosis activity, became the new communication hub in the pro‐inflammatory microenvironment, eventually contributing to the senescence of SC via the EREG‐EGFR pairs.

Our interactome analysis also suggested robust communication between effector T cells and macrophages through secreted proteins, including IFN‐γ, CCL3L1/CCL3, and TNF‐α. Previous studies have illustrated that IFN‐γ and TNF‐α promoted the expression of genes such as *CASP4*, *CASP1*, *IL1B*, *NLRP3*, *GSDMD*, *GBP1*, *GBP2*, *IRF2*, and *SERPINB1*, highly suggestive of pyroptosis activation.^[^ ^35^ ^]^ We speculated the increased number of effector T cells and elevated expression of IFN‐γ and TNF‐α in middle‐aged groups further facilitated pyroptosis activation in macrophages, exacerbating pro‐inflammatory functions.

After elucidating how these cells coordinate their functions via ligand‐receptor‐mediated communication, we speculated that this transformation in the modes of intercellular communication might be the cause of ovarian aging.

### 2.7 Pyroptotic Macrophages Promote SC Senescence

**Figure 5:**
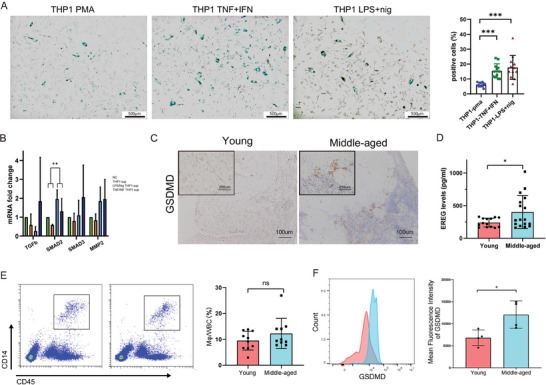
Pyroptotic macrophages promote stromal cell senescence. A) Representative images of senescence‐associated β‐galactosidase (SA‐beta‐gal) staining of stromal cells treated with the supernatant of TNF‐α/IFN‐γ‐stimulated THP1 cells, the supernatant of LPS/Nig‐stimulated THP1 cells or the supernatant of unstimulated THP1 cells (scale bar, 500 µm). Statistical analyses of SA‐beta‐gal‐positive cell rates were shown in the right image. B) The relative expression of fibrosis‐related genes in ovarian stromal cells treated with supernatant of unstimulated or stimulated THP1 cells. The results were analyzed by ANOVA. C) Representative IHC staining for GSDMD showing the elevated expression of GSDMD in the middle‐aged group (scale bar, 100 µm). The insert at top left corner of each image is the magnified image (scale bar, 250 µm). D) EREG concentration in follicular fluids measured by ELISA. E) Flow cytometric analysis of follicular fluids from IVF patients: representative flow cytometric dot plots of macrophages in follicular fluids from each group. Statistical graph of the percentages of macrophages in follicular fluids. F) Representative histogram plots and the statistical graph of the MFI of GSDMD. MFI: mean fluorescence intensity.

We subsequently sought to explore the direct effects of macrophages on SCs in vitro, based on the prediction of intercellular crosstalk in the sequencing results. Pyroptosis was successfully induced in THP1 cell as previously reported (Figure S6A, Supporting Information). Pyroptotic THP1 showed a decreased expression of *SPP1* and increased expression of *IL1B*, *EREG*, and *CASP1*, presenting a VCAN+‐macrophage‐like phenotype (Figure S6B, Supporting Information). The supernatant medium was harvested to treat human ovarian SCs. After 24 h of stimulation, the treated SCs were collected for SA‐beta‐gal staining or PCR assays. Compared with unstimulated THP1 supernatant (THP1‐pma), the supernatant of pyroptotic THP1 largely increased the number of beta‐gal positive SCs. No significant difference in the percentage of beta‐gal positive cells was observed between SCs treated with supernatant of pyroptotic THP1 induced by IFN‐γ/TNF‐α or Nig/LPS (**Figure** **5**A). However, SCs stimulated with IFN‐γ/TNF‐α or Nig/LPS showed no increase in cellular senescence (Figure S6C, Supporting Information), indicating that IFN‐γ/TNF‐α and Nig/LPS treatment accelerated SC aging through promoting the development of pyroptosis in macrophages rather than exerting a direct effect on SCs. We also proved that soluble factors produced by effector T cells (PMA and ionomycin‐induced Jurkat cells) can stimulate the secretion of IL1b in macrophages(Figure S6D, Supporting Information). Based on previous studies, these cytokines are highly likely IFN‐γ/TNF‐α.^[^ ^36^, ^37^ ^]^

Meanwhile, the expression of key genes in the fibrosis‐related pathway, such as *SMAD2*, *SMAD3*, and *MMP2* was upregulated (Figure 5B; Figure S6E, Supporting Information), reflecting increased synthesis of ECM proteins in aging SCs. In brief, pyroptotic macrophages activated the TGF‐beta signaling pathway in SCs to promote the production of ECM components and simultaneously accelerated SC senescence.

This concept of pyroptosis has been verified in clinical samples. The expression of GSDMD, the major executor of pyroptosis, was upregulated in middle‐aged ovaries by IHC staining (Figure 5C; Figure S6F, Supporting Information). Given the difficulty in obtaining extra fresh ovarian tissues, we collected follicular fluids from IVF patients of different ages for flow cytometric analysis and ELISA, which have been proposed to be the indicator of the ovarian inflammatory microenvironment. The concentration of pyroptosis‐related cytokine, EREG, the critical driving factor of SC senescence, was significantly increased in follicular fluids from elder individuals (Figure 5D). While the proportions of macrophages did not differ significantly, the mean fluorescent intensity (MFI) of GSDMD expression was significantly higher in the middle‐aged group, as shown in the histogram in Figure 5E,F.

### 2.8 Inhibition of Pyroptosis Delays Ovarian Aging and Promotes Female Health

To investigate whether the age‐related changes in cellular composition and gene expression in macrophages could explain the decline of reproductive potential, we constructed GSDMD‐/‐ mice, the executor of pyroptosis, and sought to recapitulate the impact of pyroptosis on ovarian aging.

**Figure 6:**
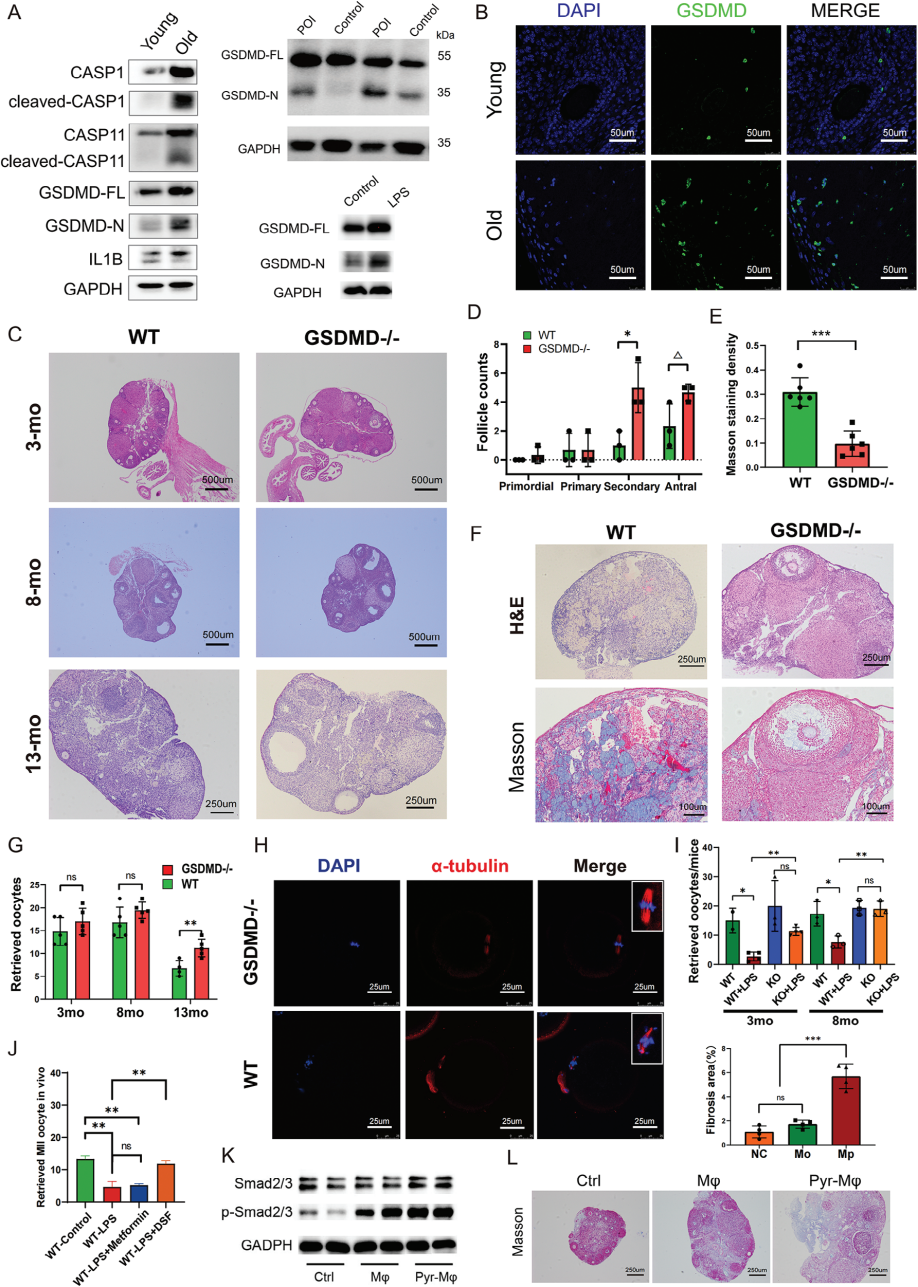
The inhibition of pyroptosis impedes ovarian aging and promotes female health. A) The expression levels of pyroptosis‐related proteins in ovaries from naturally aged (old), prematurely aged (POI) and LPS‐stimulated mice were detected by western blotting. B) Representative immunofluorescence images of GSDMD staining of ovarian sections from young and old mice, with nuclei counterstained with DAPI (scale bar, 50 µm). C) Representative HE‐stained ovarian tissue sections from mice of different genotypes at the ages of 3 months (3 mo), 8 months (8 mo), and 13 months (13 mo). D) The total number of follicles of each stage was counted and statistically analyzed. The error bars indicate SD. *p<0.05, △ shows a rising tendency. E) Quantitation analysis of Masson staining of ovarian sections from WT and GSDMD‐/‐ mice. F) H&E and Masson staining of ovarian sections from 21‐month‐old WT and GSDMD‐/‐ mice. Collagen fibers were dyed blue. G) The histogram shows the comparison of MII oocyte numbers collected from WT and GSDMD‐/‐ mice at the age ages of 3 months (3 mo), 8 months (8 mo), and 13 months (13 mo). H) Immunofluorescence images showing chromosome morphology (DAPI, blue) and microtubules (α‐tubulin, red) of the WT and GSDMD‐/‐ oocytes (scale bar, 25 µm). Higher magnification images are shown on the right. I) Histogram showing the comparison of MII oocyte numbers collected from LPS‐stimulated WT and GSDMD‐/‐ mice at the ages of 3 months and 12 months; J) The number of oocytes obtained from LPS‐stimulated mice fed a standard diet, diet with DSF, and or diet with metformin (Met). PBS treatment served as a normal control. K) Ovaries were harvested 2 days after intraovarian transplantation of PBS, Mφs, and Pyr‐ Mφs for western blot analysis of the expression of TGFB/Smad pathway components. L) Bottom: Masson staining of ovaries after culture in vitro with conditional media of macrophages (Mφ) or pyroptotic macrophages (Pyr‐Mφ) for 2 days. Upper: Statistical analysis of the percentage of fibrosis area: fibrotic area/total area. Scale bar: 250 µm.

First, we validated that pyroptosis was involved in ovarian aging, including natural aging, premature aging induced by cyclophosphamide (CTX) – busulfan (BUS) and acute ovarian function decline induced by lipopolysaccharide (LPS). the expression of GSDMD‐FL, GSDMD‐N and Caspase‐1 was examined by western blot (**Figure** **6**A; Figure S7A, Supporting Information). Consistent with our sequencing results, the percentage of F4/80+ macrophages decreased in natural aging ovaries (Figure S7B, Supporting Information) and the GSDMD+ cells accumulated in the ovarian stroma in old individuals (Figure 6B; Figure S7C, Supporting Information).

GSDMD knockout efficiency was confirmed by western blot assay (Figure S7D, Supporting Information). We then performed histological analysis of ovaries of different ages to determine whether GSDMD deletion could slow the normal ovarian aging process. We observed no significant difference in growing follicles between young WT and KO mice (3 to 8 months of age). Follicles of all stages were gradually lost with increasing age, and differences in follicle number were first observed in mice over the age of 12 months. As shown in the representative figures, no late‐growing follicles or antral follicles could be found in WT ovaries, while follicles containing a large antrum were easily observed in KO ovaries (Figure 6C). The statistical analysis of follicle numbers showed significantly increased numbers of growing follicles in KO mice at the age of 13 months (Figure 6D). The differences were further magnified with prolonged observation time. At the age of 21 months, nearly no follicle structures were observed in WT ovaries. Not only that, the Masson staining and the corresponding quantification of collagen density further suggested that the majority of the stroma in WT ovaries was filled with fibrous tissue, a phenotype that is closely related to impaired fertility. In contrast, however, the ovaries of age‐matched KO mice contained large antral follicles and corpora lutea, indicating a preserved fertility (Figure 6E,F). These results implied that follicle loss was faster in WT mice and that GSDMD deletion protected age‐related decline in ovarian reserve.

Meanwhile, we hormonally induced ovulation in young adult (3 months), middle‐aged (8 months) and aged (13 months) mice of different genotypes to compare the quantity and quality of ovulated oocytes. Similarly, we retrieved more MII oocytes from GSDMD‐/‐ mice of all ages, but these trends did not reach statistical significance until the age of 13 months (Figure 6G). Our data were similar to those of previous publications showing that the protective effect of ASC or NLRP3 depletion on follicle loss was not manifested until 12 months.^[^ ^11^ ^]^ Spindle morphology and chromosome alignment are strongly associated with oocyte quality. Most GSDMD‐/‐ MII oocytes presented typical barrel‐shaped spindles with a normal distribution of chromosomes, while meiotic defects were more prevalent in age‐matched WT mice. Representative images are shown in Figure 6H. We also performed terminal deoxynucleotidyl transferase (TdT) dUTP nick‐end labeling (TUNEL) assays and immunohistochemistry staining of PCNA to investigate the apoptosis and proliferation status in aged ovaries, but the results were not comparable across groups.

LPS was reported to activate the pyroptosis process and jeopardize the quality and quantity of obtained oocytes. Therefore, we administered LPS at a concentration of 5 mg k^−1^g to GSDMD ‐/‐ and wild‐type C57 mice to induce ovarian acute inflammation. GSDMD deletion protected mice from the sharp decline in body weight and reduction in the numbers of ovulate oocytes after LPS treatment (Figure 6I). Interestingly, the use of disulfiram (DSF), a well‐recognized pyroptosis inhibitor, partially rescued the decreased number of ovulated oocytes in LPS‐treated WT mice (Figure 6J). These results validated the hypothesis suggested by our sequencing profile, that is, pyroptosis is implicated in the ovarian aging process, and GSDMD inhibition could be a promising therapeutic strategy.

Furthermore, in vivo and ex vivo experiments were designed to explore whether the pyroptosis activation in macrophages, but not in other cells, was the cause of accelerated ovarian aging. BMDMs were isolated from WT mice and stimulated with LPS/Nig to induce pyroptosis. The supernatant medium was collected as conditional medium for in vitro ovary culture. Cells were harvested and transferred directly into the ovaries. Two days after intraovarian injection, mice were sacrificed to collect ovaries for histological examinations. Although no obvious changes in morphology were observed, western blot results showed increased phosphorylation of Smad2/3, indicating the activation of fibrosis‐related pathways (Figure 6K; Figure S7E, Supporting Information).

In vitro ovary culture was performed as previously reported,^[^ ^38^, ^39^ ^]^ and conditioned medium was added at a ratio of 1:1 to ex vivo culture medium. The morphology of the ovaries was analyzed after 2 days of culture. Treatment with supernatant from pyroptotic macrophages (Pyr‐Mφ) caused extensive tissue damage to the ovary, as demonstrated by an increase in the number of atretic follicles in the ovarian cortex and elevated collagen deposition in the ovarian medulla (Figure 6L; Figure S7F, Supporting Information). These results suggested pyroptosis activation in macrophages resulted in accelerated ovarian aging.

## 3 Discussion

Understanding the process of ovarian aging is crucial for early intervention for fertility preservation, since ovarian function exhibits a non‐appreciable decline in early 30s. Numerous studies have attempted to reveal the mechanisms underlying ovarian aging from different perspectives, a persistent chronic inflammatory microenvironment as reflected by the increased level of TNF‐α, IL6 and IL1B,^[^ ^40^ ^]^ is frequently mentioned. However, due to the limitations of analytic techniques and ovarian tissue acquisition, the cellular composition and single‐cell gene expression profiles of human ovaries of different ages have not been fully elucidated. In this study, we took advantage of ScRNA‐seq to generate a comprehensive ovarian cell atlas for women throughout the entire reproductive span. Through deducing a cell‐cell interaction network, we further identified macrophages as the central element of the immunomodulation mechanisms controlling the ovarian aging process. First, we identified nine major cell types in adult human ovaries, which approximately overlapped with previously published single‐cell profiling.^[^ ^17^, ^20^ ^]^ Second, we conducted a comparison between young and aged groups and observed a conspicuous age‐related variation within distinct cell types. More specifically, SCs, which accounted for the majority of somatic cells in our scRNA‐seq results, changed dramatically in both number and cellular status in the middle‐aged group. SCs were reported to be closely related to extracellular matrix formation, which is responsible for the ovarian fibrosis process. Previous studies have confirmed the essential role of fibrotic microenvironment in ovarian aging process by disturbing surrounding secondary follicle development and impairing oocyte release,^[^ ^41^, ^42^ ^]^ which could be reversed by metformin and antifibrosis drugs (pirfenidone and BGP‐15).^[^ ^43^, ^44^, ^45^ ^]^

We found the proportion of senescent SCs was significantly increased in middle‐aged group, with higher expression of senescence‐related and fibrosis‐related genes. Senescent SCs responded abnormally to extracellular stimuli, such as hormones or cytokines. Notably, chronic exposure to high levels of luteinizing hormone (LH)^[^ ^41^ ^]^ or androgens^[^ ^46^ ^]^ was reported to promote ovarian fibrosis. We then explored the underlying mechanisms of these changes by performing pseudotime analysis within each SC subcluster and revealed a similar immune regulatory mechanism. Immune factors, especially CD44, played a pivotal role among all SC clusters. Thus, we speculated that changes in ovarian immune microenvironment leads to the SC senescence, which contributes to the excessive production and deposition of extracellular matrix (ECM) components in the ovarian stroma, and eventually depletes the ovarian reserve.

Macrophages are key players in the ovarian immune microenvironment. Many ovarian pathological conditions, such as aging, polycystic ovary syndrome and ovarian cancer, have been reported to be related to the perturbation of the M1/M2 balance.^[^ ^27^, ^47^ ^]^ Although the exclusive nature of the M1/M2 classification has been challenged recently,^[^ ^26^ ^]^ studies based on M1/M2 model have provided abundant information. A recent study from mice illustrated a significant reduction in total macrophage number and a shift from M1 to M2 polarization in aged ovaries.^[^ ^16^ ^]^ In terms of ontogeny, it could be speculated from the bulk RNA‐seq profiles that the enhanced recruitment of circulating monocytes caused monocyte‐derived macrophages (MDMs) to become dominant in the macrophage pool. This was partially consistent with our results, although the authors failed to clarify the fate of infiltrating MDMs. Previous studies were limited by the fact that the classical M1/M2 model is not able to reflect true heterogeneity of ovarian macrophages. Here, we not only identified four macrophage subtypes by unsupervised clustering, but also described the specific markers of ovarian resident macrophages that have not been identified previously. Thereafter, we set out to determine the multiple functions of macrophage populations in the ovarian microenvironment. We first grouped the macrophages into 4 subclusters, designated as C1QC+ TRMs, HLA‐DQA1+ TRMs, SPP1+ TRMs, and VCAN+ MDMs. The C1QC+/SPP1+ tumor‐associated macrophage (TAM) dichotomy was first noted in colorectal cancer (CRC) by Lei Zhang et al^[^ ^29^ ^]^ and it was confirmed by Xiong Li et al that this dichotomy could discriminate cervical cancer patients with different prognoses.^[^ ^48^ ^]^ Thus, it was feasible to apply this ubiquitous macrophage classification in our study. C1QC+ TAMs were reported to play roles in immunosurveillance, while SPP1+ TAMs played a protumorigenic/prometastatic role in tumors. Although the macrophages in our study shared similar phenotypes with those in CRC, they performed different functions in cancer and nonneoplastic tissues, since the frequency of C1QC+ TRMs and SPP1+ TRMs in middle‐age group remained unchanged or slightly decreased, respectively, which seems contradictory to the increase in tumor incidence with age.

To explore the relationships among these clusters, we performed pseudotime analysis, which revealed that VCAN+ MDMs could give rise to the SPP1+ TRM subset and C1QC+ TRM subset, neither of which exhibited M1/M2 phenotypes. The obvious accumulation of VCAN+ MDMs and decreased frequency of SPP+/C1QC+ TRMs in the middle‐aged group led us to speculate that the transition from VCAN+ MDMs to SPP1+/C1QC+ TRMs might be impeded with age. Given that women ovulate monthly during their reproductive years, the ovary experiences a persistent wounding and healing process, and the stroma undergoes recurrent ECM remodeling. It is plausible that the persistent presence of chronic inflammation in middle‐aged ovaries promoted the infiltration of circulating monocytes, resulting in the accumulation of VCAN+ MDMs. However, for the impeded developmental process, we analyzed the differentially expressed genes (DEGs) along the pseudotime trajectory and clustered them into six expression patterns. The major expression pattern distinguishing the two branches, involved genes such as *IL1B*, *NLRP3*, *CASP4*, *SERPINA1*, and *SERPINB1*, which were highly expressed in pre‐branch (VCAN+ MDMs) and decreased in cell state 1 (SPP1+ TRMs) and increased in cell state 2 (C1QC+ TRMs). Interestingly, these genes have been reported to participate in pyroptosis, a newly discovered programmed cell death mechanism that is highly relevant to inflammation. Considering the unique functional features of macrophages, we further defined those macrophages with an enrichment of pyroptosis‐related genes as pyroptotic macrophages.

To gain a deeper insight into the regulatory roles of pyroptotic and non‐pyroptotic macrophages in the ovarian immune microenvironment, we applied ligand‐receptor pairing analysis to construct macrophage‐centric networks and revealed an age‐related switch in macrophage interactome. Non‐pyroptotic macrophages played a the pivotal immunoregulatory role by secreting SPP1, and interacting with CD44+ SCs and AREG+ T cells. These interactions contributed to the maintenance of “young” SCs, with limited collagen fiber production, and meanwhile, restricted the shift from AREG+ T cells to effector T cells. However, long‐term exposure to sterile inflammation in middle‐aged ovaries activated pyroptosis pathways, leading to aberrant macrophage development. Finally, the macrophage pool was dominated by VCAN+ MDMs. The accumulation of pyroptotic macrophages in the middle‐aged group greatly aggravated ovarian inflammation through their interactions with effector T cells and EGFR+ SCs via the production of IL1B and EREG. In turn, effector T cells promoted the aging of SCs and activated the pyroptosis pathway in macrophages by secreting proteins including IFN‐γ, CCL3L1/CCL3 and TNF‐α. This vicious cycle would accelerate fibrosis, undermine follicular development and ultimately result in reduced fertility throughout the reproductive lifespan.

Fibrosis has been recognized as an important factor contributing to tumorigenesis. A recent study on postmenopausal women revealed a correlation between aged fibrotic ovaries and M2 polarization in macrophages, promoting the formation of a tumor‐permissive niche.^[^ ^49^ ^]^ The immunosuppressive microenvironment in ovarian cancer is closely related to the production of TGFBI by macrophages. The accumulation of macrophages in a proinflammatory status, mainly VCAN+ MDMs, despite their retained immune surveillance functions, would lead to a persistent chronic inflammatory environment in aged ovaries, ultimately resulting in fibrosis, which is permissive to tumorigenesis. Thus, the fact that the incidence of ovarian malignant disorders is relatively low during reproductive age but increases after menopause is probably due to the accumulation of chronic inflammation triggered by macrophages.^[^ ^50^ ^]^ However, the features of ovarian resident macrophages in postmenopausal women were not discussed in detail in our study. Further in‐depth studies will be needed to verify the hypothesis that whether the specific alterations in macrophages and their subsequent effects might potentially contribute to the elevated occurrence of ovarian malignancies in postmenopausal women. In addition to bioinformatics analysis, we performed in vitro cell experiments to confirm the putative cellular communication among macrophages, SCs and TC. GSDMD expression, as assessed by IHC, is upregulated in ovarian stroma from middle‐age women. Moreover, we observed an elevated expression of GSDMD and pyroptosis‐related cytokines, EREG, in follicular fluids from elder IVF patients, reflecting an alteration in ovarian tissues. As a direct microenvironment for oocyte development, pyroptosis activation in follicular fluids might have profound impacts on oocyte quality, which required further researches.

In view of these findings, we constructed GSDMD knockout mice, which provided a clean genetic background enabling us to unambiguously identify the functions of pyroptotic macrophages in ovarian aging process. Pyroptosis was universally activated in diverse ovarian aging models, and these aging processes could be impeded by both genetic inhibition of GSDMD and pyroptosis inhibitors.

Nevertheless, there were several limitations in this study. For ethical reasons, it is not possible to obtain ovarian tissues from healthy females with no necessary clinical reasons for surgery. This has long been a key factor limiting ovarian research in humans, and our study is not an exception. The human samples involved in this study were normal parts of ovary tissues kindly donated by patients undergoing surgery for ovarian cysts or endometriosis, but with grossly normal ovarian function. As such, we could not guarantee that there are no differences between these tissues and fully healthy ovarian tissues. However, to prevent misleading results due to possible sampling bias and obtain a compelling conclusion, we not only conducted combined analysis with public data, but also performed substantial experimental verification in multiple cell culture and animal models.

In summary, our work successfully provided a comprehensive ovarian cell atlas dissecting age‐related changes in the cell population and cell‐type‐specific genes in reproductive females. It is the first time that the subtype and phenotype of ovarian macrophages, and how they coordinate different immune responses in the ovarian microenvironment is described in detail. More importantly, we proposed and experimentally demonstrated a pyroptotic‐macrophage‐involved immune mechanisms in the ovarian aging process. Despite the study limitations, our findings largely enrich the existing knowledge related to the “inflamm‐aging” theory. We believe that the advances in understanding of ovarian aging mechanisms provided in our study could help researchers develop anti‐aging therapeutic strategies and suggest that pyroptosis might be the next potential therapeutic target for delaying the ovarian aging process.

## 4 Experimental Section

### Ethics Statement

This research was approved by the Medical Ethical Committee of the Sixth Affiliated Hospital, Sun Yat‐sen University (2019SZZX‐010). All patients provided written informed consent before biopsy. The study includes 7 patients (27–42 years) admitted to the reproductive center mainly due to male factor infertility. Detailed characteristics of clinical data for each case were summarized in supplementary table 1. Ovarian tissues (5×5×1 mm) biopsies were collected during surgery and then fragmented for single‐cell suspension preparation and histological evaluation.

### Dissociation of The Ovarian Cortex into Single‐Cell Suspension

The ovarian cortex tissue was cut into small pieces in Dulbecco's Modified Eagle Medium (DMEM, Gibco) and immersed in dissociation buffer containing 1 mg ml^−1^ Collagenase II (Sigma–Aldrich), 1 mg ml^−1^ Collagenase IV (Sigma–Aldrich), 3 mmol L^−1^ CaCl2, 0.1 mg ml^−1^ DNase I (Sigma–Aldrich) and 1 mg ml^−1^ dispase (Sigma–Aldrich), and incubated for 30–50 mins at 37 °C. Enzymatic digestion was terminated by DMEM supplemented with 10% fetal bovine serum (FBS, Gibco). Samples were filtered through 70 and 40 µm cell strainer to collect the single‐cell suspensions. After centrifugation at 300 g for 5 mins at 4 °C, the cells were re‐suspended at a concentration in phosphate buffered saline‐0.1% Tween 20 with 0.1% bovine serum albumin. The cell viability was measured using acridine orange and propidium iodide staining.

### ScRNA‐Seq of Ovarian Cells

Single‐cell suspension was loaded onto one channel of the Chromium Single Cell B Chip Kit (10x Genomics, 1 000 073). The Chromium Single Cell 3′ GEM, Library & Gel Bead Kit v3 (10x Genomics, 1 000 075) was used for single‐cell bar coding, cDNA synthesis and library preparation, following manufacturer's instructions. Spiked‐in samples were sequenced on Illumina Hiseq Xten PE150 platform (paired‐end 150 bp).

### Retrospective Analysis

This retrospective analysis included IVF/ICSI cycles for male factor infertility conducted in the reproductive center of the Sixth Affiliated Hospital, Sun Yat‐sen University between January 2019 and April 2020. In total, 347 women aged from 21 to 44 years old were recruited, 42 of whom were above 35 years old.

### Animal Experiments

All animal studies were reviewed and approved by the Animal Ethics Committee of Sixth Affiliated Hospital, Sun Yat‐sen University (IACUC‐2021051401). The female wild type C57BL/6 were purchased from Beijing Vital River Laboratory Animal Technology Co. Ltd. (Beijing, China) and Gsdmd‐/‐ mice were produced by GemPharmatech Co. Ltd (Jiangsu, China). All mice were bred at a temperature of 23–25°C with 12 h light/dark cycle, supplied with food and water ad libitum.

### Establishment of Mouse Premature Ovarian Insufficiency (POI) Model

1) POI mouse model was established by intraperitoneal injection of cyclophosphamide (120 mg k^−1^g) and busulfan (50 mg k^−1^g) once, according to previously reported. 2) Lipopolysaccharide (LPS, 5 µg g^−1^, Biotopped Life Sciences, L2630‐10 mg) was injected intraperitoneally to induce acute inflammation. The WT and KO mice were injected intraperitoneally with lipopolysaccharide (LPS, 5 µg g^−1^, Biotopped Life Sciences, L2630‐10 mg) 24 h after PMSG administration.

### Pyroptosis Inhibitor Intervention

To achieve proper intervention with pyroptosis inhibitors, the metformin and disulfiram (DSF) diet were produced by milling metformin (5 g kg^−1^, Apexbio, B1970) and DSF (1 g kg^−1^, Apexbio, A4015) with standard rodent diet.^[^ ^51^ ^]^ Ten‐day period of this dietary intervention was performed before oocyte retrieval.

### Oocyte Retrieval

Superovulation of mice was induced by pregnant mare serum gonadotropin (PMSG) (Ningbo Second Hormone Factory) and human chorionic gonadotropin (HCG) (Ningbo Second Hormone Factory). Briefly, mice were injected with 5–10 IU PMSG and 48 h later, with 5–10 IU HCG and then the ovulated oocytes were retrieved from oviducts. The oocytes were further examined and recorded after removing the cumulus cells by pipetting in 0.1% hyaluronidase.

### Histological Analysis

H&E staining, Masson staining and immunohistochemistry staining were performed as previously published. Collagen contents were stained blue and the other stained red. The ratio of blue area was quantitated by Image J (1.8.0).

### Immunofluorescence Staining

The ovarian tissues were fixed in 4% paraformaldehyde solution, then dehydrated and embedded in paraffin. The tissues were sectioned at 5 µm. The slides were dewaxed in xylene and rehydrated through graded ethanol concentrations. Antigen retrieval was performed in citrate solution and then were blocked with 3% BSA for 1 h. After incubating with the primary antibody (CD3, Affinity Biosciences, DF6594; CD14, ABclonal, A19011; DDX4, Abcam, ab27591, GSDMD, Affinity, AF4012) overnight at 4 °C, the slides were incubated with appropriate secondary antibody (CY3 goat anti‐mouse IgG, EarthOx, E031610‐01; CY3 goat anti‐rabbit IgG,EarthOx, E031620‐01; DyLight 488 goat anti‐rabbit IgG, EarthOx, E032220‐01; Alexa Fluor 647 goat anti‐mouse, Abcam, ab150115) for 1 h at room temperature, followed by staining with 4′,6‐diamidino‐2‐phenylindole (DAPI, meilunbio) and imaged with Leica TCS‐SP8 Confocal Laser Scanning Microscope (Leica Microsystems).

### Ex Vivo Ovary Culture

The ovaries were isolated from mice of 6 weeks and the periovarian adipose tissue were removed under stereomicroscope. The ovaries were cut in half with razor blade and tissue blocks were randomly divided into each group. The ovaries were cultured on a Millipore Insert (PICMORG50, Milipore) in the 6‐well culture plate (NEST, Biotechnology) containing the indicated culture medium. The Basic medium was Dulbecco's modified Eagle's medium/nutrient mixture F‐12 (DMEM/F‐12; Thermo Fisher Scientific) supplemented with penicillin‐streptomycin and 1% insulin‐transferrin‐selenium (ITS). Conditional culture medium was prepared by mixing supernatant of macrophages under different treatments with basic culture medium in a volume ratio of 1:1. Then the ovaries were incubated at 37 °C with 5% CO_2_ and saturated humidity.

### Cell Culture

Primary human ovarian stromal cells (purchased from iCell Bioscience Inc), THP1 cells, and Jurkat T cells (purchased from FuHeng Biology) were cultured under appropriate conditions. THP1 cells (FuHeng Biology, FH0112) were primed with 1 ng ml^−1^ phorbol myristate acetate (PMA, Sigma–Aldrich, P1585) for 24 h to differentiate to macrophages. The macrophages were treated as follows, respectively: 1) 100 ng ml^−1^ interferon‐γ (IFN‐γ, Peprotech, 300–02) for 24 h; 2) 50 ng ml^−1^ tumor necrosis factor‐α (TNF‐α, Peprotech, 300–01A) for 24 h; 3) 50 ng ml^−1^ TNF‐α for 24 h followed by 100 ng ml^−1^ IFN‐γ for 24 h; 4) 100 ng ml^−1^ LPS for 4 h with 10 µM nigericin (Sigma–Aldrich, 481 990) for 2 h; 5) 100 ng ml^−1^ IFN‐γ for 24 h followed by 100 ng ml^−1^ LPS for 4 h with 10 uM nigericin for 2 h; 6) 50 ng ml^−1^ TNF‐α for 24 h followed by 100 ng ml^−1^ IFN‐γ for 24 h, then 100 ng ml^−1^ LPS for 4 h with 10 µM nigericin for 2 h; 7) 1 µg ml^−1^ LPS transfection (FuGENE HD transfection Reagent, Promega, E2312); 8) 100 ng ml^−1^ IFN‐γ for 24 h followed by 1 µg ml^−1^ LPS transfection; 9) 50 ng ml^−1^ TNF‐α for 24 h followed by 100 ng ml^−1^ IFN‐γ for 24 h and 1 µg ml^−1^ LPS transfection. Cells were collected for Western blot and the culture medium was collected for IL1β detection.

Human T cell line Jurkat cells (FuHeng Biology, FH0878) were activated with 50 ng ml^−1^ PMA and 300 ng ml^−1^ ionomycin (AbMole, 56092‐82‐1) for 6 h and cultured without stimulation for 4 h in RMPI 1640 medium (BasalMedia). The supernatants of Jurkat cells were cultured with THP1‐differentiated macrophages for 24 h and the supernatants of THP1 cells were collected for IL1B detection.

### Senescence‐Associated‐Beta‐Galactosidase (SA‐Beta‐Gal) Staining

SA‐beta‐gal staining was performed on primary human ovarian stromal cells (iCell Bioscience Inc, HUM‐iCell‐f001) using Senescence β‐Galactosidase Staining Kit (G1580, Solarbio, G1580). In brief, human ovarian stromal cells were seeded in 6 well plates and treated with 1 µg ml^−1^ SPP1 (Novoprotein, C544), 100 ng ml^−1^ EGF (Peprotech, AF‐100‐15), and supernatants of pyroptotic macrophages for 24 h. To obtain the supernatants of pyroptotic macrophages, PMA‐differentiated THP1 cells were treated IFN‐γ (100 ng ml^−1^) and TNF‐α (50 ng ml^−1^) for 24 h or LPS (1 µg µl^−1^) for 4 h followed by nigericin (10 µM) for 2 h.

### Flow Cytometry

The number of macrophages and the relative expression of GSDMD were determined by flow cytometry. In brief, follicular fluids without obvious blood stain were collected from discarded clinical specimens and then centrifuged at 500 g for 5 mins at 4 °C. Cells were washed two times with ice‐cold PBS and blocked in 2% BSA before staining with antibodies. To avoid non‐specific staining, the anti‐GSDMDC1 antibodies (NBP2‐80427, NOVUS) were first labelled with FITC using Antibody Labeling Kit. Other antibodies included APC anti‐human CD45 (368 511, Biolegend) and PE anti‐human CD14 antibodies (301 805, Biolegend). One blank tube and 2 single‐dye adjustment compensation tubes were used as controls. Single cells were first gated using FSC‐A/SSC‐A, followed by FSC‐H/FSC‐A to remove doublet. Then gate was set on CD14+ CD45+ cells, and the MFI of GSDMD was determined by flow cytometry. Assays were performed on CytoFLEX Flow Cytometer (Beckman, Inc) and analyzed by FlowJo (10.8.1)

### Quantitative Real‐Time PCR (qRT‐PCR)

Total RNA from young and old mice ovary was extracted using RNeasy Micro kit (Qiagen, 74 004). And cDNA was synthesized with a reverse transcription kit (Biosharp, BL699A). Quantitative RT‐PCR was performed with the Roche LightCycler480 Real‐Time PCR system using the RealStar Power SYBR Mixture (Genstar, A311). The critical threshold cycle (Ct) value was determined for each reaction, which was transformed into relative quantification data using the 2^−ΔΔCt^ method. The housekeeping gene β‐actin was used as an internal control.

### Western Blots

Ovaries fragments or cells were lysed in RIPA buffer with protease inhibitor (Apexbio) and phosphatase inhibitor (Apexbio) for 10 mins and centrifuged at 12,000 g for 10 mins at 4 °C. The supernatant mixed with SDS‐PAGE sample buffer (GenStar) was denatured at 100 °C for 10 mins. Denatured protein was separated on 10–15% SDS‐PAGE gel and transferred to Polyvinylidene Fluoride membrane. Blocked with 5% non‐fat dry milk, the membranes were incubated with primary antibodies of GSDMD (Abcam, ab219800), IL1beta (Abcam, ab205924), caspase 1 (AdipoGen, AG‐20B‐0042‐C100), caspase 11 (AdipoGen, AG‐20B‐0060‐C100) and GAPDH (Affinity, AF7021) overnight at 4 °C and the corresponding secondary antibodies for 1 h at room temperature. The blots were imaged with ChemiDoc imaging system (Bio‐Rad).

### ELISA

Concentrations of AMH in mouse serum and IL1β in culture medium were detected by ELISA kits (Cusabio) according to the manufacturer's instructions.

### ScRNA‐Seq Data Analysis

For the scRNA‐seq, sequenced reads were quantified using the Cell Ranger (version 3.1.0) and aligned against the Genome Reference Consortium Human Build 38 (GRCh38) by STAR software (Dobin et al., 2013). ScRNA‐seq data were loaded to R version 4.0.2 and processed with Seurat package version 3.6.0. Genes fulfilled the following criteria were selected for further analysis: gene count per cell >200 and <4000, unique molecular identifiers (UMI) count per cell < 60000, percentage of mitochondrial genes < 15%. Graph‐based cluster analysis was performed using Cell Ranger (version 3.1). Uniform manifold approximation and projection (UMAP) dimensionality reduction was performed using Seurat (version 3.6.0). FindAllMarkers function in (Seurat version 3.6.0) was adopted to identify the marker gene of each cluster. GeneOntology enrichment analysis of marker genes were performed by using ClusterProfiler (Version 3.14.3). Gene expression values were used to generate heatmaps. Cell clusters and cells were annotated according to the HPCA/BLUEENCODE annotation library of SingleR (Version 1.0.1). Pseudotime analysis were conducted by using R package Monocle2. Branch expression analysis modeling (BEAM) was performed to find genes that were regulated in a branch‐dependent way. Differentially expressed genes (DEGs) was defined as genes with absolute fold change >1.5 and p value <0.05. Gene set enrichment analysis (GSEA) was conducted based on these DEGs using the OmicStudio tools at https://www.omicstudio.cn/tool. RNA velocity analysis was performed on velocyto.R (Version 0.6). CellphoneDB version 2.2.7 was applied to infer the cell‐cell communication networks and ligand‐receptor pairs, and plot the heatmaps. Pyroptosis pathway activity of individual cells was scored by AUCell package.^[^ ^30^ ^]^ The pyroptosis pathway gene set from MSigDB (https://www.gsea‐msigdb.org/gsea/msigdb/human/geneset/REACTOME_PYROPTOSIS.html) was abtained.

### Statistical Analysis

Statistical analysis was conducted using GraphPad Prism version 8. Experimental outcomes were described as the average ± s.d. of 3 biological replicates. Two sided Kruskal‐Wallis test was applied to determine the significant differences among groups. P<0.05 was regarded statistically significant.

## Conflict of Interest

The authors declare no conflict of interest.

## Author Contributions

C.Z., Q.G., J.L. (Jiayu Lin), and M.W. contributed equally to this work. X.L. and H.Z. conceived, organized, and supervised the overall study. C.Z. was responsible for executing experiments, analyzing the data and performing bioinformatics analysis. This manuscript was written by Q.G. and M.W. with the help from all the other authors. J.L. (Jiayu Lin), Y.X., Q.L., J.L. (Jiawen Liu), T.W. and Y.Z. performed all of the cell and animal experiments. Z.Z., Y.L. and X.L. recruited the patients and carried out the biopsies. S.H. and S.W. provided valuable suggestions. This manuscript was revised by X.L. and C.Z. All authors reviewed the manuscript, checked the data and approved the submitted version.

## Supporting information

Supporting Information

## Data Availability

The data that support the findings of this study are available on request from the corresponding author. The data are not publicly available due to privacy or ethical restrictions.
