# Supplementary material for: Single‐Cell Atlas of Human Ovaries Reveals The Role Of The Pyroptotic Macrophage in Ovarian Aging
Source: Adv Sci (Weinh). 2023 Nov 30;11(4):2305175. doi: 10.1002/advs.202305175 (PMC10811476; doi:10.1002/advs.202305175)
Supplement: Supplementary file 1 — Supporting Information [file ADVS-11-2305175-s001.pdf]

ADVANCED SCIENCE
Open Access

# Supporting Information

for *Adv. Sci.*, DOI 10.1002/advs.202305175

Single-Cell Atlas of Human Ovaries Reveals The Role Of The Pyroptotic Macrophage in Ovarian Aging

*Chuanchuan Zhou, Qi Guo, Jiayu Lin, Meng Wang, Zhi Zeng, Yujie Li, Xiaolan Li, Yuting Xiang, Qiqi Liang, Jiawen Liu, Taibao Wu, Yanyan Zeng, Shanyang He, Sanfeng Wang, Haitao Zeng* and Xiaoyan Liang**

# Supporting Information

## Title: Single-cell atlas of human ovaries reveals the role of the pyroptotic macrophage in ovarian aging

*Chuanchuan Zhou*[†]*, Qi Guo*[†]*, Jiayu Lin*[†]*, Meng Wang*[†]*, Zhi Zeng, Yujie Li, Xiaolan Li, Yuting Xiang, Qiqi Liang, Jiawen Liu, Taibao Wu, Yanyan Zeng, Shanyang He, Sanfeng Wang, Haitao Zeng*,Xiaoyan Liang*.*

**Tables**

**Supplementary Table 1. Characteristic and applications of human ovarian tissue.**

| Number | Age (years) | Main Diagnosis | Number | Age (years) | Main Diagnosis |
|---|---|---|---|---|---|
| 1* | 27 | Ovarian endometriosis | 17# | 31 | Ovarian endometriosis |
| 2* | 27 | Ovarian endometriosis | 18# | 31 | Ovarian endometriosis |
| 3* | 29 | Ovarian endometriosis | 19# | 32 | Ovarian endometriosis |
| 4* | 33 | Ovarian endometriosis | 20# | 34 | Ovarian endometriosis |
| 5* | 37 | Ovarian endometriosis | 21# | 34 | Ovarian endometriosis |
| 6* | 42 | Ovarian endometriosis | 22# | 36 | Ovarian endometriosis |
| 7* | 42 | Ovarian cystadenoma | 23# | 40 | Ovarian endometriosis |
| 8# | 18 | Mature teratoma | 24# | 46 | Leiomyoma |
| 9# | 21 | Mature teratoma | 25# | 46 | Adenomyosis |
| 10# | 24 | Ovarian cystadenoma | 26# | 47 | Cervical cancer |
| 11# | 24 | Mature teratoma | 27# | 48 | Ovarian cystadenoma |
| 12# | 26 | Ovarian endometriosis | 28# | 51 | Leiomyoma |
| 13# | 27 | Ovarian endometriosis | 29# | 51 | Ovarian fibrothecoma |
| 14# | 28 | Mature teratoma | 30# | 51 | Leiomyoma |
| 15# | 30 | Mature teratoma | 31# | 56 | Leiomyoma |
| 16# | 31 | Kidney cancer for fertitlity preservation | | | |

*For Single-cell RNA sequencing; #For Immunofluorescence staining

**Figure.S1**

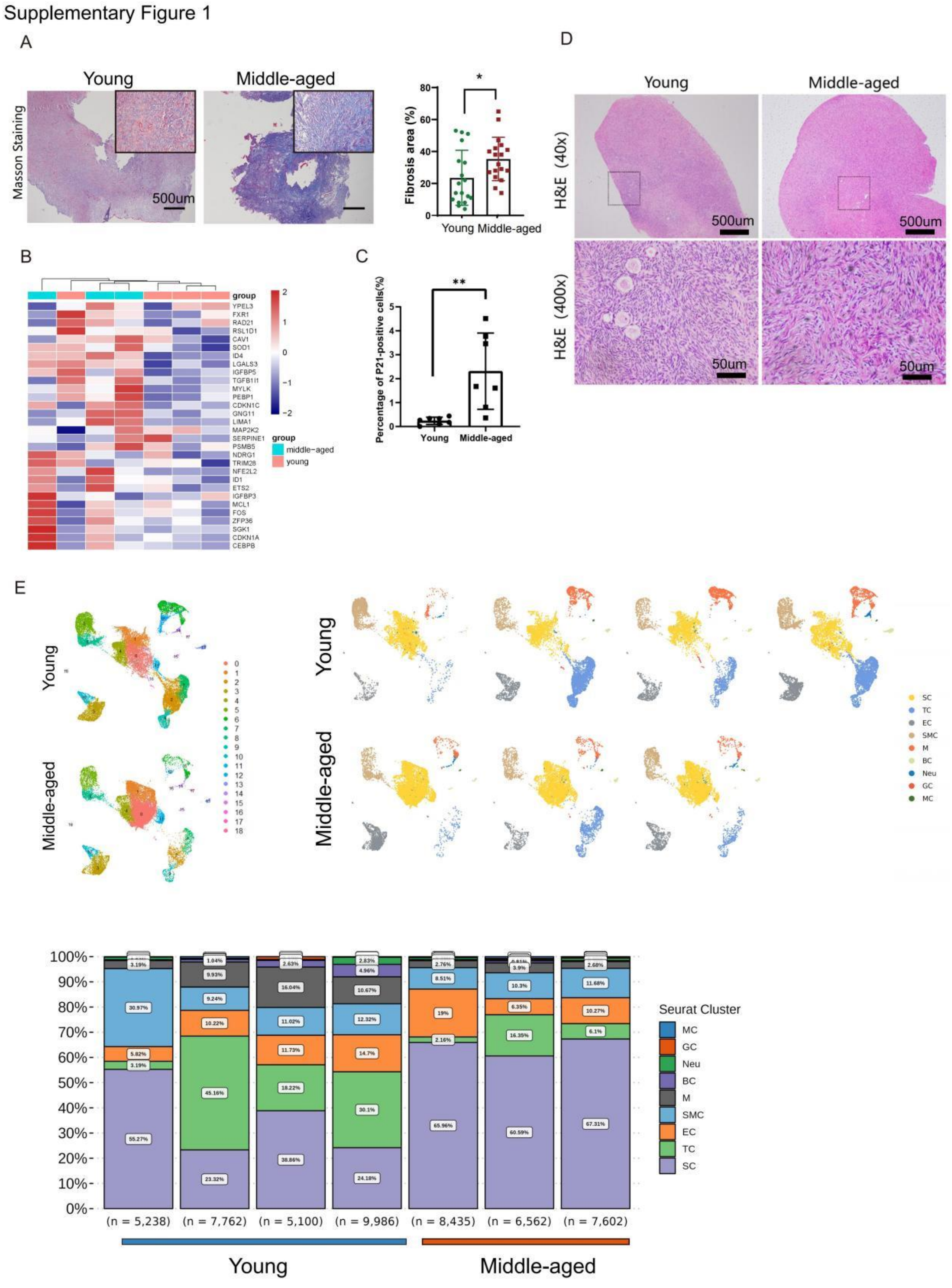


(A) Representative Masson staining of ovarian sections from young and middle-aged women, and the collagen fibers were dyed blue. The fibrosis area was calculated as the area of fibrosis to the

total area. A total of 30 sections from 10 individuals were analyzed and the corresponding statistical results were showed in the right.

(B) The heatmap of the expression levels of the aging-related markers mentioned in CellAge databases.

(C) Statistical analysis of P21-positive cells in each group of IHC (n=14).

(D) The typical H&E stainning of ovarian from young and middle-age sequencing individuals, showing the different density of follicles. Upper panel is low magnification (40x) and lower is high magnification (400x).

(E) The comparison of cellular composition among individuals. Left image: UMAP plots showing the cluster distributions in the 4 and 3 pooled samples. Right image: Each Umap plot of annotated cell types represented one sample. Bottom: Bar graphs representing the composition ratio of each cell type from each individual. n: number of cells in each sample.

## Figure.S2

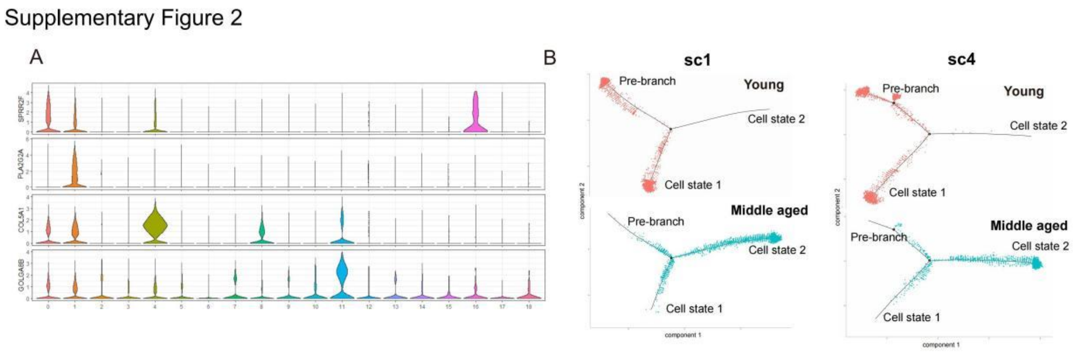


(A) Violin plots showing the expression levels of stromal cell signature genes in all the clusters.

(B) Pseudotime analysis were performed in sc1 and sc4, respectively. Comparison of the distribution of stromal cells (sc1 and sc4) from the young and middle-aged groups along the pseudotime trajectories.

## Figure.S3

Supplementary Figure 3

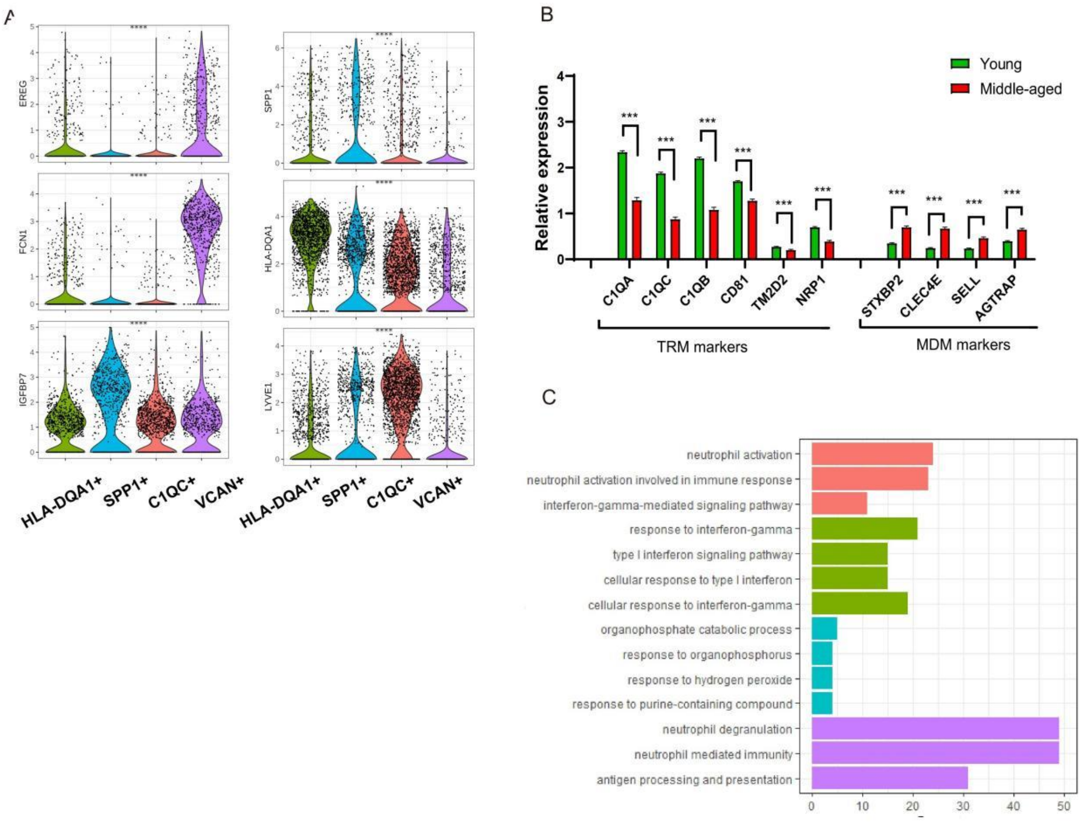


(A)The violin plots showing the expression of marker genes, EREG, FCN1, IGFBP7, SPP1, HLA-DQA1 and LYVE1 in each macrophage subcluster.

(B) Expression of marker genes of TRM and MDM in the two groups.

(C) The functional enrichment analysis of age-related genes in each subcluster.

**Figure.S4**

Supplementary figure 4

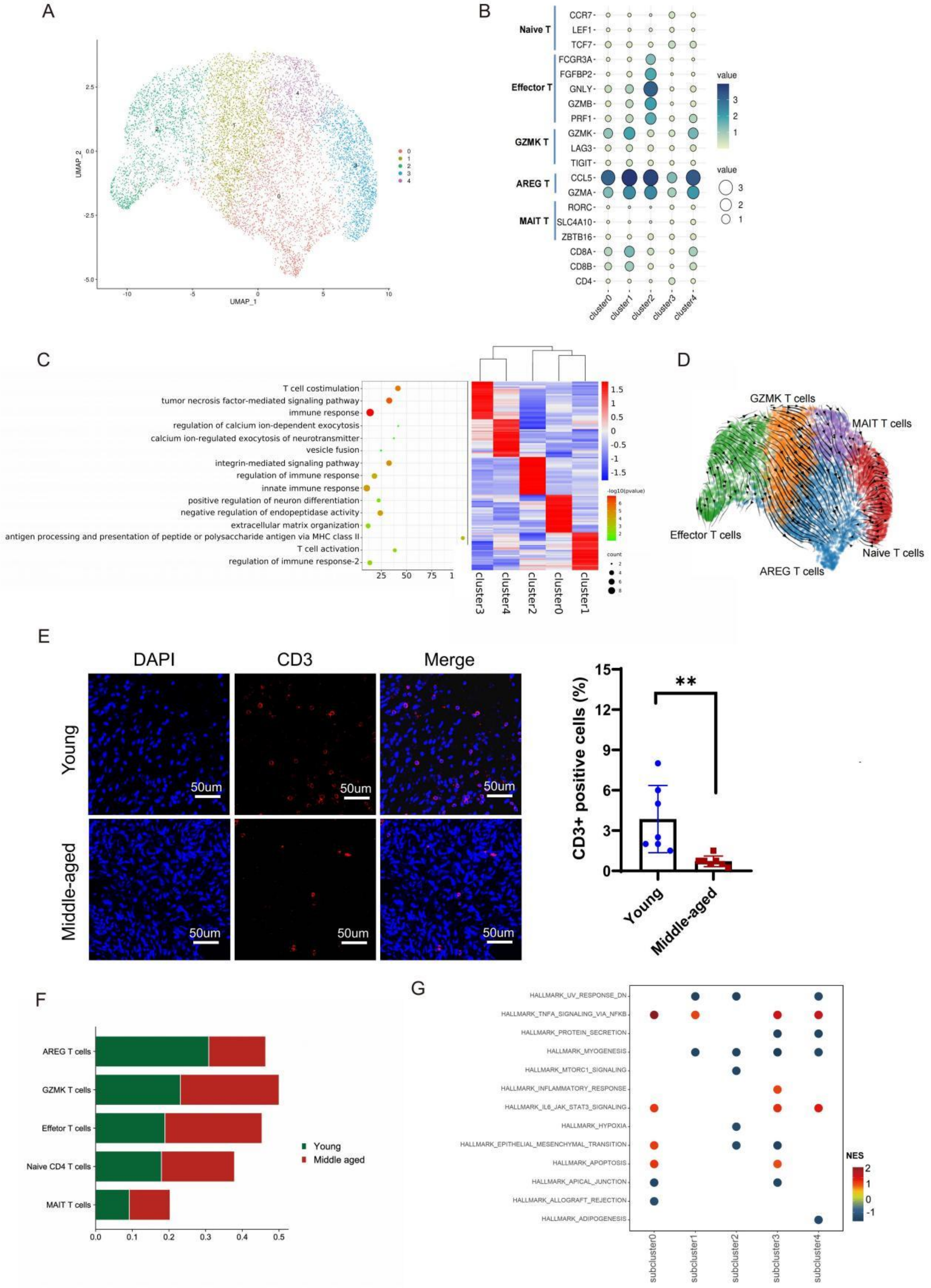


(A) UMAP plot showing the results of re-clustering of T cell populations.

(B) The bubble plot showing the expression of canonical T-cell signature genes in each cluster.

(C) Enrichment of the KEGG pathways of each T cell subtype.

(D) The results of RNA velocity analysis showing the trajectory.

(E) Immunostaining of young (upper panel) and middle-aged (lower panel) ovarian sections for CD3. Bar: 50um. The number of CD3 positive cells was counted and displayed in the right statistical graph.
(F) Bar plot showing the proportions of each T cell subset in the young and the middle-aged group.
(G) GSEA analysis of the age-related DEGs in each T cell subcluster. NES, normalized enrichment score.

## Figure.S5. The interactome revealed by ligand-receptor pair analysis.

Supplementary Figure 5

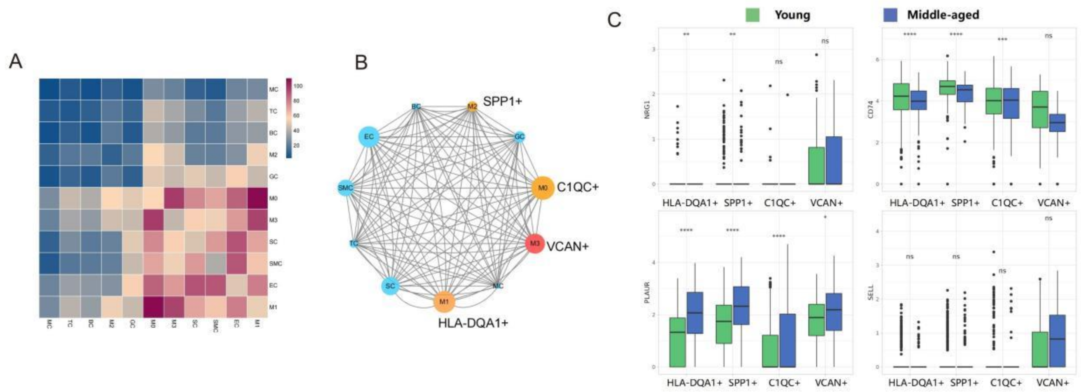


(A) Heatmap ofcell-cell interactions based on the numbers of ligand-receptor pairs.
(B) Interaction network diagram of all ovarian somatic cell types. Circle size indicates the number of connections with other cell types.
(C) Boxplots showing the differential expression of selected ligands in macrophage subclusters between the young and middle-aged groups.

## Figure. S6. Pyroptotic macrophages promote stromal cell senescence.

Supplementary Figure 6

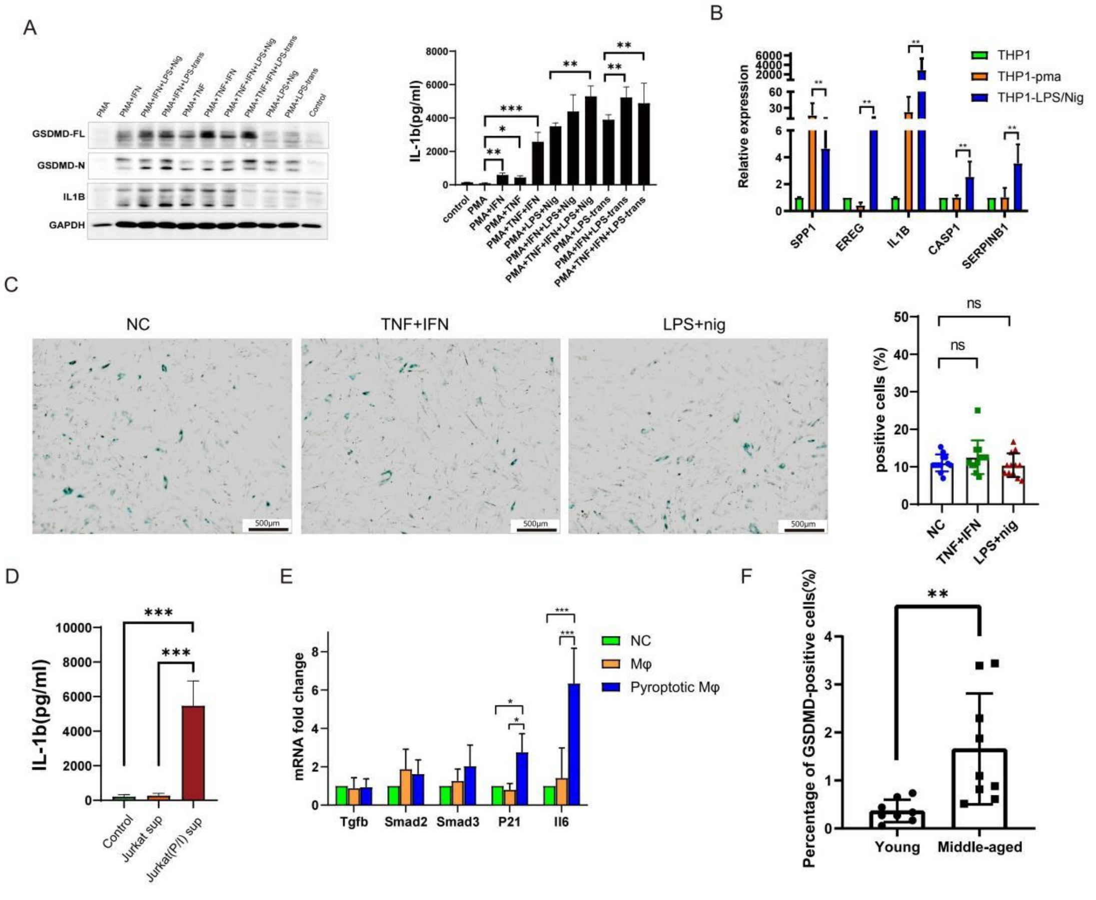


(A)THP1 cells were treated as indicated, GSDMD-FL, cleaved GSDMD and IL1β was measured by WB (left). IL1β in cell culture medium was measured by ELISA (right).

(B) Relative expression of SPP1, EREG, IL1B, CASP1 and SERPINB1 in THP1 cells, PMA-primed THP1 cells and LPS/Nig-stimulated THP1 cells.

(C) Representative images of senescence-associated β-galactosidase (SA-beta-gal) staining of stromal cells treated with IFN, TNF and LPS/Nig. Statistical analyses of SA-beta-gal-positive cell rates were shown in the right image.

(D) ELISA assay for the IL1β detection of untimulated (Jurkat sup) and Effector-T-cell stimulated THP1 cells (Jurkat P/I sup). Effector T cells were induced in Jurkat T cells by PMA and ionomycin. The supernatant was harvested to stimulate THP1 cells.

(E) The relative expression of fibrosis-related genes in murine ovarian stromal cells treated with supernatant of unstimulated (Mφ) and pyroptotic macrophages (Pyroptotic Mφ). The results were analyzed by ANOVA.

(F) Statistical analysis of GSDMD-positive cells in each group of IHC (n=18).

**Figure. S7. The inhibition of pyroptosis impedes ovarian aging and promotes female health.**

Supplementary Figure 7

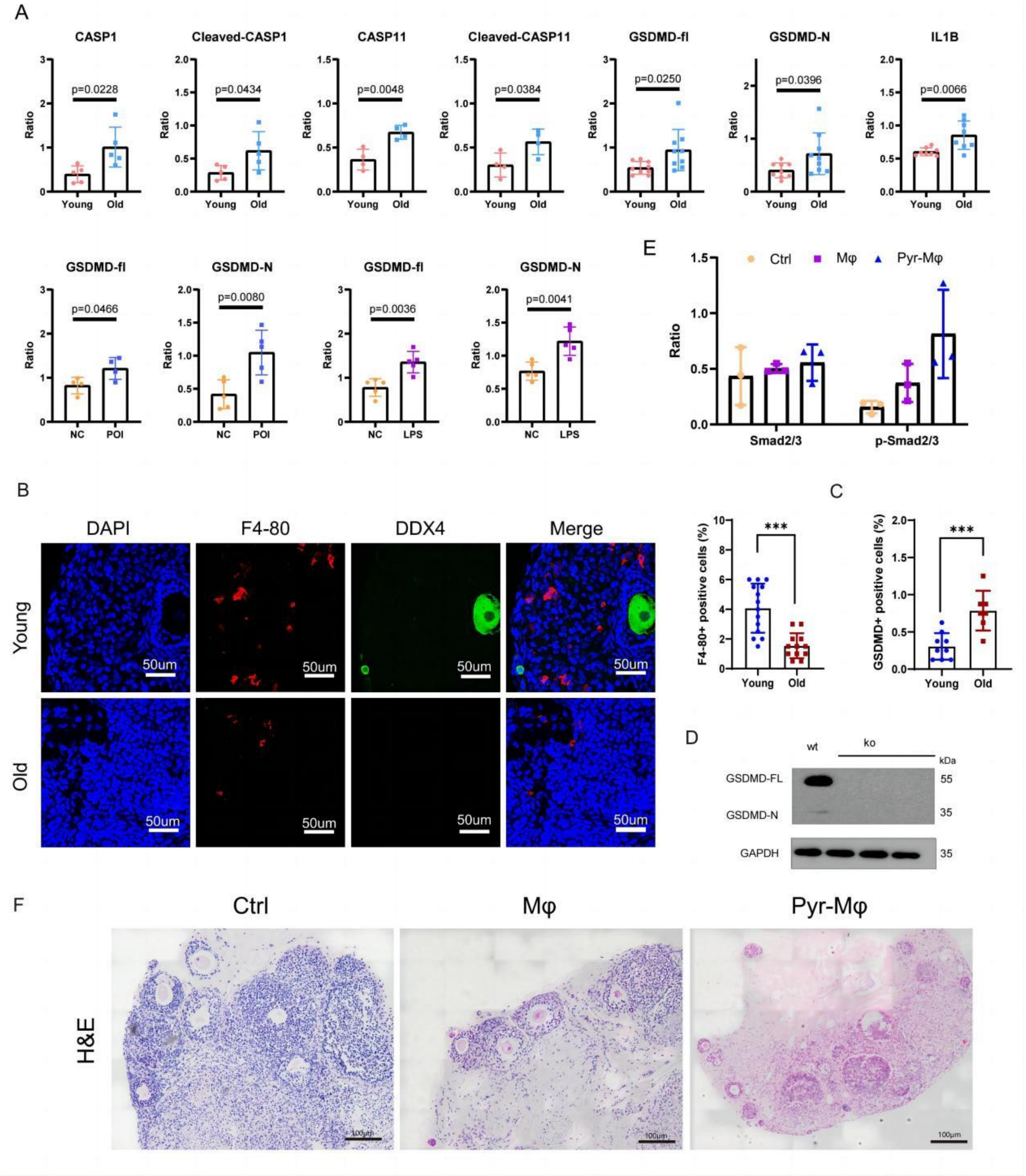


(A) The quantification of WB bands was conducted by ImageJ and the statistical analysis is associated with Figrue.6A.

(B) Representative immunofluorescence images of F4/80 staining of ovarian sections from young and old mice. The number of F4/80+ positive cells was counted and displayed in the right statistical graph.

(C) The number of GSDMD positive cells was counted and displayed in the statistical graph.

(D) Verification of knockout efficiency by WB.

(E) The quantification of WB bands was conducted by ImageJ and the statistical analysis is associated with Figrue.6K.

(F) Histological analysis of the murine ovaries after culture in vitro for 2 days.
